# Quantifying arousal and awareness in altered states of consciousness using interpretable deep learning

**DOI:** 10.1038/s41467-022-28451-0

**Published:** 2022-02-25

**Authors:** Minji Lee, Leandro R. D. Sanz, Alice Barra, Audrey Wolff, Jaakko O. Nieminen, Melanie Boly, Mario Rosanova, Silvia Casarotto, Olivier Bodart, Jitka Annen, Aurore Thibaut, Rajanikant Panda, Vincent Bonhomme, Marcello Massimini, Giulio Tononi, Steven Laureys, Olivia Gosseries, Seong-Whan Lee

**Affiliations:** 1 Department of Brain and Cognitive Engineering, Korea University, Seoul, Republic of Korea; 2 Coma Science Group, GIGA-Consciousness, GIGA Research Center, University of Liège, Liège, Belgium; 3 Centre du Cerveau², University Hospital of Liège, Liège, Belgium; 4 Wisconsin Institute for Sleep and Consciousness, Department of Psychiatry, University of Wisconsin, Madison, USA; 5 Department of Neuroscience and Biomedical Engineering, Aalto University School of Science, Espoo, Finland; 6 Department of Neurology, University of Wisconsin, Madison, WI USA; 7 Department of Biomedical and Clinical Sciences “L. Sacco”, University of Milan, Milan, Italy; 8 Fondazione Europea di Ricerca Biomedica, FERB Onlus, Milan, Italy; 9 IRCCS Fondazione Don Carlo Gnocchi ONLUS, Milan, Italy; 10 Department of Anesthesia and Intensive Care Medicine, University Hospital of Liège, Liège, Belgium; 11 University Department of Anesthesia and Intensive Care Medicine, CHR Citadelle, Liège, Belgium; 12 Anesthesia and Intensive Care Laboratory, GIGA-Consciousness, GIGA Research Center, University of Liège, Liège, Belgium; 13 Department of Psychology, University of Wisconsin, Madison, WI USA; 14 Department of Artificial Intelligence, Korea University, Seoul, Republic of Korea

**Keywords:** Sleep, Predictive markers, Disorders of consciousness

## Abstract

Consciousness can be defined by two components: arousal (wakefulness) and awareness (subjective experience). However, neurophysiological consciousness metrics able to disentangle between these components have not been reported. Here, we propose an explainable consciousness indicator (ECI) using deep learning to disentangle the components of consciousness. We employ electroencephalographic (EEG) responses to transcranial magnetic stimulation under various conditions, including sleep (*n* = 6), general anesthesia (*n* = 16), and severe brain injury (*n* = 34). We also test our framework using resting-state EEG under general anesthesia (*n* = 15) and severe brain injury (*n* = 34). ECI simultaneously quantifies arousal and awareness under physiological, pharmacological, and pathological conditions. Particularly, ketamine-induced anesthesia and rapid eye movement sleep with low arousal and high awareness are clearly distinguished from other states. In addition, parietal regions appear most relevant for quantifying arousal and awareness. This indicator provides insights into the neural correlates of altered states of consciousness.

## Abstract (web summary)

The authors propose an explainable consciousness indicator using deep learning to quantify arousal and awareness under sleep, anesthesia, and in patients with disorders of consciousness.

## Introduction

Responsiveness is often thought to reflect consciousness, and for a long time, unresponsiveness was considered as a surrogate of unconsciousness. However, consciousness and responsiveness are two different concepts^1^. Consciousness is considered to be absent during sleep or anesthesia, but in certain instances, subjective experience can still occur (e.g., dreaming)^2,3^. Similarly, consciousness has been described as a result of both arousal and awareness components^4^. Arousal refers to the overall state of alertness (or wakefulness). In contrast, awareness refers to the subjective experience, such as perceiving a blue triangle versus a red circle^5^. Typically, at the clinical level, arousal is indicated by the opening of the eyes, and awareness is inferred by the ability to follow commands.

Various levels of consciousness exist in physiological, pharmacological, and pathological modifications of consciousness (Table 1). In non-rapid eye movement (NREM) sleep with no subsequent reports of subjective experiences, both arousal and awareness are low. However, in rapid eye movement (REM) sleep with dreams, arousal is low but awareness can reach high levels^5,6^. Under general anesthesia, propofol and xenon predominantly induce states similar to NREM sleep without subjective experiences, whereas ketamine induces dream-like experiences similar to REM sleep with subjective reports upon awakening^3,7^. Additionally, recent findings suggest that a minority of patients under anesthesia may also be conscious of their environment during a surgical procedure, which is referred to as connected consciousness. These patients exhibit the ability to follow commands using the isolated forearm technique^8^, without recollection upon awakening. Unresponsive wakefulness syndrome (UWS) describes patients who recover with their eyes open, but only demonstrate reflex behaviors, while patients in a minimally conscious state (MCS) present reproducible non-reflex movements^9^. In both UWS and MCS patients, arousal is high; however, unlike UWS, MCS patients also show signs of awareness that can be considered as a sign of volitional behavior^10,11^. However, certain UWS patients (assessed several times with the Coma Recovery Scale-Revised (CRS-R)^12^ may present brain activity similar to MCS patients (e.g., high brain metabolism measured by positron emission tomography). This peculiar state has been termed non-behavioral MCS or MCS*^13,14,15^.

**Table 1:** Schematic representation of different states of consciousness according to low or high arousal and awareness: the plus sign indicates high arousal or awareness, whereas the minus sign indicates low arousal or awareness.

Condition	State	Arousal	Awareness
Physiology	Healthy wakefulness	+	+
	REM sleep with dreams	−	+
	NREM sleep without dreams	−	−
Pharmacology	Anesthesia induced with ketamine	−	+
	Anesthesia induced with propofol or xenon	−	−
Pathology	MCS	+	+
	MCS*	+	+
	UWS	+	−

*REM* rapid eye movement, *NREM* non-rapid eye movement, *MCS* minimally conscious state, MCS* non-behavioral MCS, *UWS* unresponsive wakefulness syndrome.

Note that the anesthesia-induced with propofol and xenon mentioned here does not include the use of the isolated forearm technique.

An effective measure of consciousness, labeled perturbational complexity index (PCI), was developed from electroencephalographic (EEG) responses to direct and noninvasive cortical perturbation with transcranial magnetic stimulation (TMS)^16^. PCI quantifies the complexity of deterministic patterns of significant cortical activation evoked by TMS. This index was validated in a large benchmark population to derive an empirical cutoff (PCI* = 0.31) that reliably discriminates between unconsciousness (PCI_max_ ≤ PCI*: NREM sleep; midazolam-, propofol- and xenon-induced anesthesia) and consciousness (PCI_max_ > PCI*: REM sleep; wakefulness; ketamine-induced anesthesia; and conscious brain-injured patients)^17^. However, PCI cannot discriminate REM sleep or ketamine-induced anesthesia from healthy wakefulness. In addition, multiple trials are required to compute PCI^16,17^. A few studies have attempted to develop an objective measure of consciousness from resting-state EEG brain activity^3,4,18,19^. Interestingly, the spectral exponent, which quantifies the slope of power spectral density of resting-state EEG activity, is another measure of consciousness that is highly correlated with PCI and allows distinguishing between ketamine and propofol or xenon-induced anesthesia^3^. In addition, when low- (1–20 Hz) and high-band (20–40 Hz) spectral exponents are jointly considered, ketamine-induced anesthesia can be distinguished from wakefulness, and the xenon and propofol-induced anesthesia conditions are partially superimposed in the spectral exponent; consequently, these are difficult to distinguish^3^. Recently, a decrease in high-frequency oscillations and an increase in low-frequency power in the primary sensory, motor, and visual cortices were observed during REM sleep when compared to healthy wakefulness^20^. The quantification of the spectral slope between 30 and 50 Hz was also proposed for discriminating REM sleep from healthy wakefulness^4^. However, this measure did not differentiate REM from NREM sleep; thus, it distinguishes between different arousal levels but not awareness levels. Therefore, an alternative measure to simultaneously disentangle the two components of consciousness, requiring fewer trials, would be a valuable and necessary tool.

The classical neurophysiological approach for calculating PCI, power spectral density, and spectral exponent relies on many epochs to improve the reliability of statistical estimates of these indices^21^. However, these methods are only suitable for investigating the averaged brain states and they can only clarify general neurophysiological aspects. Machine learning (ML) allows decoding and identifying specific brain states and discriminating them from unrelated brain signals, even in a single trial in real-time^22^. This can potentially transform statistical results at the group level into individual predictions^9^. A deep neural network, which is a popular approach in ML, has been employed to classify or predict brain states using EEG data^23^. Particularly, a convolutional neural network (CNN) is the most extensively used technique in deep learning and has proven to be effective in the classification of EEG data^24^. However, a CNN has the drawback that it cannot provide information on why it made a particular prediction^25^. Recently, layer-wise relevance propagation (LRP) has successfully demonstrated why classifiers such as CNNs have made a specific decision^26^. Specifically, the relevance score resulting from the LRP indicates the contribution of each input variable to the classification or prediction decision. Thus, a high score in a particular area of an input variable implies that the classifier has made the classification or prediction using this feature. For example, neurophysiological data suggest that the left motor region is activated during right-hand motor imagery^27^. The LRP indicates that the neural network classifies EEG data as right-hand motor imagery because of the activity of the left motor region^28^. Therefore, the relevance score was higher in the left motor region than in other regions. Thus, it is possible to interpret the neurophysiological phenomena underlying the decisions of CNNs using LRP.

In this work, we develop a metric, called the explainable consciousness indicator (ECI), to simultaneously quantify the two components of consciousness—arousal and awareness—using CNN. The processed time-series EEG data were used as an input of the CNN. Unlike PCI, which relies on source modeling and permutation-based statistical analysis, ECI used event-related potentials at the sensor level for spatiotemporal dynamics and ML approaches. For a generalized model, we used the leave-one-participant-out (LOPO) approach for transfer learning, which is a type of ML that transfers information to a new participant not included in the training phase^24,27^. The proposed indicator is a 2D value consisting of indicators of arousal (ECI^aro^) and awareness (ECI^awa^). First, we used TMS–EEG data collected from healthy participants during NREM sleep with no subjective experience, REM sleep with subjective experience, and healthy wakefulness to consider each component of consciousness (i.e., low/high arousal and low/high awareness) with the aim to analyze correlations between the proposed ECI and the three states, namely NREM, REM, and wakefulness. Next, we measured ECI using TMS–EEG data collected under general anesthesia with ketamine, propofol, and xenon, again with the aim to measure correlation with these three anesthetics. Before anesthesia, TMS–EEG data were also recorded during healthy wakefulness. Upon awakening, healthy participants reported conscious experience during ketamine-induced anesthesia and no conscious experience during propofol- and xenon-induced anesthesia. Finally, TMS–EEG data were collected from patients with disorders of consciousness (DoC), which includes patients diagnosed as UWS and MCS patients. We hypothesized that our proposed ECI can clearly distinguish between the two components of consciousness under physiological, pharmacological, and pathological conditions.

To verify the proposed indicator, we next compared ECI^awa^ with PCI, which is a reliable index for consciousness. Then, we applied ECI to additional resting-state EEG data acquired in the anesthetized participants and patients with DoC. We hypothesize that if CNN can learn characteristics related to consciousness, it could calculate ECI accurately even without TMS in the proposed framework. In terms of clinical applicability, it is important to use the classifier from the previous LOPO training of the old data to classify the new data (without additional training). Therefore, we computed ECI in patients with DoC using a hold-out approach^29^, where training data and evaluation data are arbitrarily divided, instead of cross-validation. Finally, we investigated why the classifier generated these decisions using LRP to interpret ECI^30^. We show that proposed ECI using interpretable deep learning distinguishes arousal and awareness between normal consciousness, sleep, anesthesia, and patients with DoC. Furthermore, we show that the parietal region is most closely related to quantifying arousal and awareness in altered states of consciousness.

## Results

### Overview of the calculation of ECI

**Fig. 1:**
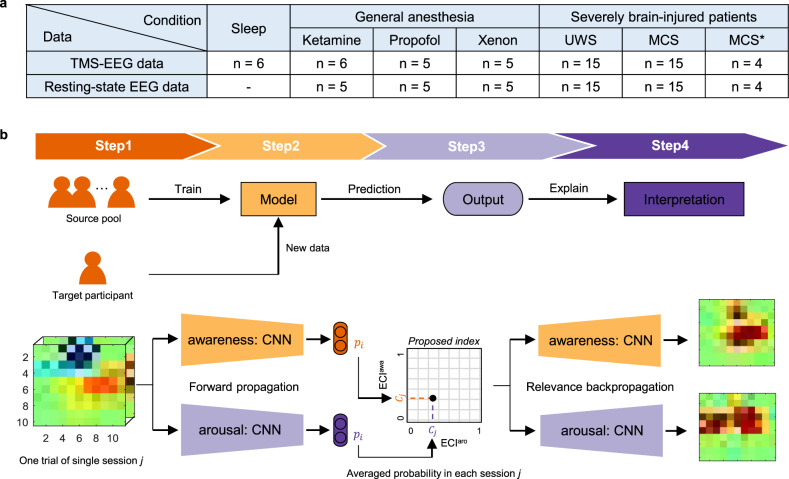
Overview of the study. **a** Data description. The same participants participated in transcranial magnetic stimulation-induced electroencephalography (TMS–EEG) and resting-state electroencephalography (EEG) measurements. The sleep condition did not include resting-state EEG, and one participant under ketamine-mediated anesthesia was missing in resting-state EEG. **b** Schematic framework for determining the explainable consciousness indicator (ECI). In step 1, raw EEG signals were converted into a spatio-spectral or spatiotemporal 3D matrix. In step 2, the converted 3D feature was used on a convolutional neural network in the two components of consciousness: arousal and awareness. In each arousal and awareness state, the EEG data were trained as two classes (low versus high). For example, for awareness, rapid eye movement (REM) sleep with subjective experience (i.e., dreaming) and healthy wakefulness belong to the same class in terms of high awareness; however, for arousal, non-rapid eye movement (NREM) with no subjective experience and REM sleep with subjective experience belong to the same class in terms of low arousal. The output ${p}_{i}$ indicates the probability in the trial *i* of arousal and awareness. For the training and test phase, we used the leave-one participant-out approach as transfer learning. Therefore, the EEG data in the source pool were used for training and the data of target participants was predicted for arousal or awareness. The source pool contains data corresponding to the source domain except for the target participant. In step 3, the interclass probability for each arousal and awareness was averaged for calculating ECI in each session *j*. The averaged probability ${C}_{j}$ is ECI^aro^ and ECI^awa^ on the *x-* and *y*-axes, respectively. Therefore, we represented the 2D consciousness indicator for the two components of consciousness. In the final step, we checked which brain signals the model has learned and why it made such a decision using layer-wise relevance propagation (LRP). Through this step, we could interpret the proposed indicator. ECI^awa^ = ECI in awareness component; ECI^aro^ = ECI in arousal component.

We used TMS–EEG data in three conditions: (i) sleep, (ii) general anesthesia, and (iii) severely brain-injured patients (Fig. 1a). Figure 1b shows the framework for calculating ECI to distinguish between low and high states in each arousal and awareness. To explore the optimal input and classifier, we compared the single-trial classification performance in each component (see Supplementary Notes 1–2, Supplementary Figs. 1–3, and Supplementary Tables 1–3). For an input in the classifier, we converted time-series EEG data to 3D data (2D meshes according to the spatial information + 1D vector according to temporal information). The CNN model was used to distinguish low from high state in each arousal and awareness. Next, the interclass probability in the new target participant was calculated using the trained model. Finally, ECI was measured by averaging the probability of a high state in each arousal and awareness over a single session.

To improve the classification performance, we trained the models with domain transfer learning, which uses the knowledge learned in one domain to improve generalization, based on similarity in the sense of pooling the training data across domains (Supplementary Fig. 4). When referring to domain transfer learning using the EEG signals in this study, the domain refers to the clinical condition in which the EEG signals were acquired. Precisely, three domains were considered here: sleep, anesthesia, and patients with DoC. In domain transfer learning, the target domain indicates a state that contains a single session for calculating ECI, whereas the source domain indicates those sessions that are included in the training phase. Therefore, the information (knowledge) trained in the source domain is applied when testing the target domain, and this is described as the transfer of learned information for domain transfer learning. In the LOPO approach, the data from all the participants in the source domain, except for the target participant, were used for training (Supplementary Fig. 3b). Note that the data for training and testing did not overlap. We used all three domains to classify high and low states in both arousal and awareness (Table 2). Consequently, the classification performance was higher when training with a closer domain (see Supplementary Notes 3–4 and Supplementary Table 4). Therefore, we trained these domains together when calculating ECI in sleep and anesthesia domains, but trained the DoC domain along with the anesthesia domain when calculating ECI in patients with DoC.

**Table 2:** Averaged single-trial classification accuracy (%) in physiological, pharmacological, and pathological conditions for TMS–EEG: this represents the accuracy ± standard deviation.

Target domain	Source domain	Arousal	Awareness
Sleep	Sleep	87.79 ± 2.50	91.95 ± 4.74
	Sleep + Ane	87.23 ± 2.99	89.96 ± 5.48
	Sleep + DoC	80.73 ± 5.05	89.60 ± 4.26
	Sleep + Ane + DoC	84.01 ± 3.46	91.14 ± 4.29
Ane	Ane	79.01 ± 10.61	80.20 ± 10.06
	Ane + Sleep	82.58 ± 6.92	87.78 ± 6.46
	Ane + DoC	69.99 ± 11.89	82.22 ± 10.66
	Ane + Sleep + DoC	72.68 ± 17.22	85.61 ± 9.09
DoC	DoC	–	75.84 ± 14.71
	DoC + Sleep	75.94 ± 18.14	79.44 ± 15.51
	DoC + Ane	83.12 ± 12.79	75.30 ± 11.99
	DoC + Sleep + Ane	66.29 ± 19.02	78.78 ± 12.98

*Ane* anesthesia domain, *DoC* patients with disorders of consciousness domain.

The target domain implies the condition with the target participant to be tested for calculating explainable consciousness indicator (ECI) using convolutional neural network (CNN) with spatiotemporal information, and the source domain implies the conditions included in training for learning classifiers.

Then, we applied ECI to resting-state EEG data in two conditions: (i) general anesthesia and (ii) severely brain-injured patients (Fig. 1a). Similarly, we classified low and high states in resting-state EEG data for arousal and awareness using domain transfer learning (Table 3). This result was similar to the classification performance of TMS–EEG data, considering that there was no resting-state EEG during sleep in the same participants (see Supplementary Note 4). Based on the results of domain transfer learning, we used only the anesthesia domain for calculating ECI under anesthesia; however, the DoC and anesthesia domains were used for calculating ECI in patients with DoC.

**Table 3:** Averaged single-trial classification accuracy (%) in pharmacological and pathological conditions for resting-state EEG: this represents the accuracy ± standard deviation.

Target domain	Source domain	Arousal	Awareness
Ane	Ane	89.91 ± 8.01	90.14 ± 6.96
	Ane + DoC	73.33 ± 14.25	88.62 ± 8.83
DoC	DoC	–	73.03 ± 17.26
	DoC + Ane	86.04 ± 12.35	73.68 ± 15.42

The target domain implies the condition with the target participant to be tested for calculating ECI using CNN with spatiotemporal information, and the source domain implies the conditions to be included in training for learning classifiers.

### ECI in TMS combined with electroencephalography

**Fig. 2:**
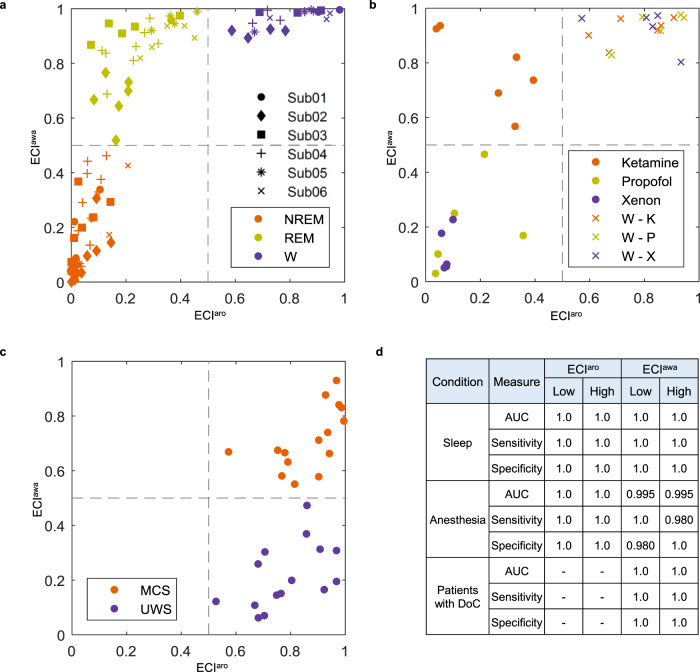
Characteristics of ECI in TMS–EEG. In ECI, the symbols show the average ECI value of each transcranial magnetic stimulation (TMS) session, and the gray dashed lines indicate the optimal cutoff (0.5) dividing the space into high and low states of ECI. **a** In sleep and normal wakefulness, we depicted P01–P06 by circles, diamonds, squares, plus signs, asterisks, and cross signs, respectively. Orange indicates NREM sleep with no subjective experience; copper indicates REM sleep with subjective experience (i.e., dreaming); moreover, purple indicates normal wakefulness. **b** In anesthesia and wakefulness before anesthesia, the orange, copper, and purple dots indicate the use of ketamine, propofol, and xenon, respectively. In addition, cross markers indicate normal wakefulness before each anesthetic. **c** In patients with disorders of consciousness, the orange and purple dots indicate patients in the minimally conscious state (MCS) and with unresponsive wakefulness syndrome (UWS), respectively. **d** In each condition, the classification performance for ECI^aro^ and ECI^awa^ was measured. *W* normal wakefulness; W – K = healthy wakefulness before ketamine; W – P = healthy wakefulness before propofol; W – X = healthy wakefulness before xenon.

Figure 2a shows ECI for each TMS session during sleep and wakefulness. This is a 2D indicator, ranging from 0 (low) to 1 (high) for both arousal and awareness. The cutoff was set to 0.5 for both arousal and awareness, as it is the mean probability for the two-class classification (low versus high). ECI in NREM sleep showed low arousal and awareness, whereas REM sleep had low arousal with high awareness. ECI in healthy wakefulness had both high arousal and awareness. We performed a receiver operating characteristic (ROC) curve analysis and determined that the area under the curve (AUC), sensitivity, and specificity for low and high arousal when using ECI were all equal to 1.0. The AUC, sensitivity, and specificity for low awareness were 0.995, 1.0, and 0.980, respectively, whereas, for high awareness, values of 0.995 for AUC, 0.980 for sensitivity, and 1.0 for specificity were obtained with ECI (Fig. 2d).

We measured ECI using three anesthetic drugs (ketamine, propofol, and xenon) and wakefulness before anesthesia (Fig. 2b). ECI in ketamine-induced anesthesia demonstrated low arousal and high awareness, whereas propofol- and xenon-induced anesthesia showed low arousal and awareness. Also, all periods of wakefulness showed high arousal and awareness. As a result of the ROC analysis, classification using ECI achieved an AUC, sensitivity, and specificity of 1.0 for all parameters for both arousal and awareness (Fig. 2d).

In patients with DoC, ECI indicated high arousal and awareness in MCS patients and high arousal and low awareness in UWS patients (Fig. 2c). AUC, sensitivity, and specificity of ECI were 1.0 for both high and low states of awareness (Fig. 2d). ROC analysis for arousal was not conducted because both UWS and MCS patients were considered to have high arousal. We additionally applied ECI to four MCS* patients; these cases were not included in the training phase. Similar to patients with DoC, anesthesia and DoC domains were selected as source domains in the training phase. For the MCS* patients, ECI successfully predicted high arousal and awareness, as expected (Supplementary Fig. 5).

### Relationship with PCI

We calculated PCI in TMS–EEG sessions that had at least 80 trials with healthy participants under sleep, anesthesia, and brain-injury conditions. PCI in all three conditions was consistent with the optimal cutoff (0.31^16,17^) that maximizes the accuracy of the distinction between consciousness and unconsciousness in a benchmark population. For ECI, the optimal cutoff of 0.5 perfectly distinguished low or high states of arousal and awareness in the physiological, pharmacological, and pathological conditions (see Supplementary Note 5).

**Fig. 3:**
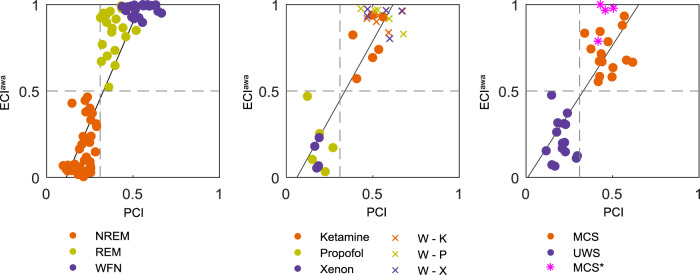
Correlation between ECI^awa^ and PCI from TMS–EEG. During sleep and healthy wakefulness (left), under anesthesia, and wakefulness before anesthesia (middle), and in severely brain-injured patients (right), ECI^awa^ was compared to PCI. The gray horizontal and vertical dashed lines represent the optimal cutoff of ECI^awa^ and PCI to discriminate between low and high awareness, respectively. The solid lines represent linear fits to the data. W healthy wakefulness, W – K healthy wakefulness before ketamine, W – P healthy wakefulness before propofol, W – X healthy wakefulness before xenon, MCS patients in a minimally conscious state, UWS patients with unresponsive wakefulness syndrome, MCS* non-behavioral MCS.

We investigated the relationship between ECI^awa^ and PCI (Fig. 3). During sleep, a positive correlation between ECI^awa^ and PCI was observed (*r* = 0.872, *p* < 0.001). Similarly, ECI^awa^ during anesthesia and in brain-injured patients showed a strong correlation with PCI (anesthesia: *r* = 0.885, *p* < 0.001; brain injury: *r* = 0.770, *p* < 0.001). ECI^awa^ and PCI, therefore, matched for all states.

### ECI in resting-state electroencephalography

**Fig. 4:**
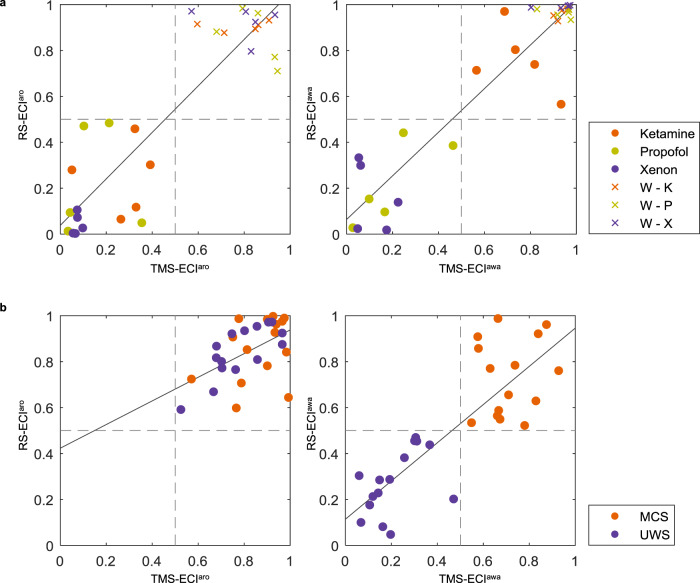
Relationship between ECI using TMS–EEG and resting-state EEG. The *x-axis* represents ECI using TMS–EEG, and the *y*-axis represents ECI using resting-state EEG for arousal (left) and awareness (right). The gray dashed lines indicate the optimal cutoff (0.5) dividing the space into high and low states of ECI. The solid lines represent linear fits to the data. **a** In anesthesia and wakefulness before anesthetics, the orange, copper, and purple dots indicate the use of ketamine, propofol, and xenon, respectively. In addition, cross markers indicate normal wakefulness before each anesthetic. **b** In patients with disorders of consciousness, the orange and purple dots indicate MCS and UWS patients, respectively.

ECI results using resting-state EEG data were similar to those when using TMS–EEG results (Fig. 4). In anesthesia, ECI using resting-state EEG statistically correlated with ECI using TMS–EEG (ECI^aro^: *r* = 0.848, *p* < 0.001; ECI^awa^: *r* = 0.938, *p* < 0.001). Similarly, in patients with DoC, there was a positive correlation (ECI^aro^: *r* = 0.534, *p* = 002; ECI^awa^: *r* = 0.832, *p* < 0.001). Similarly, we applied this method to four MCS* patients to verify ECI. As expected, similar to TMS–EEG results, accurate predictions were obtained in four MCS* patients (Supplementary Fig. 5).

### The practicality of calculating ECI

**Fig. 5:**
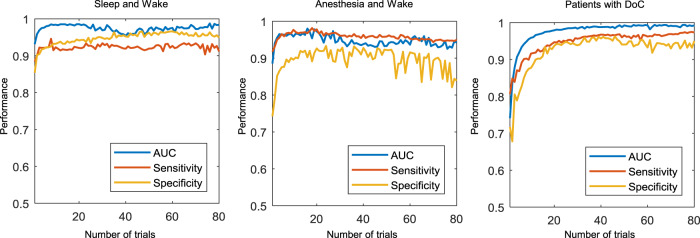
Performance of ECI^awa^ according to the number of trials in the ECI calculation. During sleep and healthy wakefulness (left), under anesthesia and wakefulness before anesthesia (middle), and in severely brain-injured patients (right). The area under the curve, sensitivity, and specificity were measured for calculating ECI^awa^ when going from single trials to 80 trials.

We explored the possibility of calculating ECI with a limited number of trials during sleep and wakefulness. The classification performance was compared through the calculation of ECI from a single trial up to the standard number of trials (i.e., 80 trials similar to PCI^16^) (Fig. 5). In the sleep and healthy wakefulness conditions, the performance reached a specificity of 0.853, a sensitivity of 0.884, and an AUC of 0.931 when using single trials (based on one TMS pulse), but from 2 trials, it was above 0.9 for specificity, sensitivity, and AUC. In the anesthesia and patients with DoC conditions, the detailed performance from 1 to 80 trials is shown in Supplementary Note 6.

**Fig. 6:**
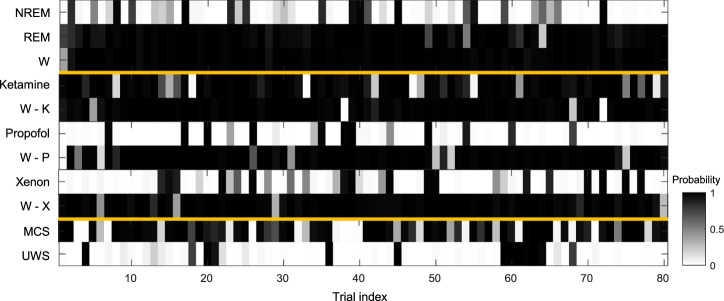
Interclass probability in the representative participant for ECI^awa^. We depicted the probability of a representative participant (P01) in all conditions (sleep, anesthesia, DoC). P01 was the first participant of each list, randomly chosen. Each colored box indicates the probability that the corresponding trial is considered as high awareness in each participant. If it was a perfect prediction in one trial, during sleep and healthy wakefulness, NREM sleep with no subjective experience (low awareness) has a probability of less than 0.5, whereas REM sleep and normal wakefulness (high awareness) have probabilities of more than 0.5. Under anesthesia and wakefulness, ketamine and wakefulness before anesthesia are high awareness, whereas propofol and xenon have low awareness. MCS and UWS patients have high and low awareness, respectively.

Figure 6 shows the possibility of awareness being high for the first participant in each of these conditions: sleep, anesthesia (ketamine, propofol, and xenon), and patients with DoC (UWS and MCS). For instance, as NREM sleep is considered to have low awareness, in P01, it was correctly predicted when the probability in a single trial was less than 0.5. Notably, NREM sleep showed that 17 trials were incorrectly predicted with a probability higher than 0.5. In addition, 1 out of 80 trials in both REM sleep and healthy wakefulness showed a value less than 0.5 and were incorrectly predicted. This indicates that ECI can be predicted as somewhat low or high, even in a single trial. There was a clear spatiotemporal difference between correct and incorrect trials only in parietal regions (Supplementary Fig. 6). No significant differences between both types of trials were observed over frontal and temporal regions. However, in parietal regions, TMS-evoked potentials at 350–400 ms were significantly higher in incorrect trials than in correct trials. In a single trial, these different patterns resulted in misprediction. Nevertheless, the effect of the incorrect trials (i.e., a failure to predict) was eliminated because ECI was calculated by averaging the interclass probability in a single trial. The probability in a single trial is shown in Supplementary Figs. 7–9 for other participants in all conditions.

To demonstrate that the method can be easily applied to a new set of patients (without additional training) to identify their state of consciousness in a clinical setting, we computed ECI using the hold-out approach. The dataset in the patients with DoC was split between the training and evaluation sets with respective ratios of 0.75 and 0.25. In other words, two MCS patients and five UWS patients were completely excluded from training, and their ECI was calculated. Consequently, ECIs using conventional LOPO and the hold-out approaches showed high positive correlation (ECI^aro^: *r* = 0.702, *p* = 005; ECI^awa^: *r* = 0.886, *p* < 0.001) (Supplementary Figure 10). In both TMS–EEG and resting-state EEG, the ECI using the hold-out method indicated high arousal and awareness in MCS patients, whereas high arousal and low awareness in UWS patients. This was consistent with 0.5 typical cutoffs. These results show that the proposed method generalizes to new data without retraining the classifier.

### Interpretation for calculating ECI

**Fig. 7:**
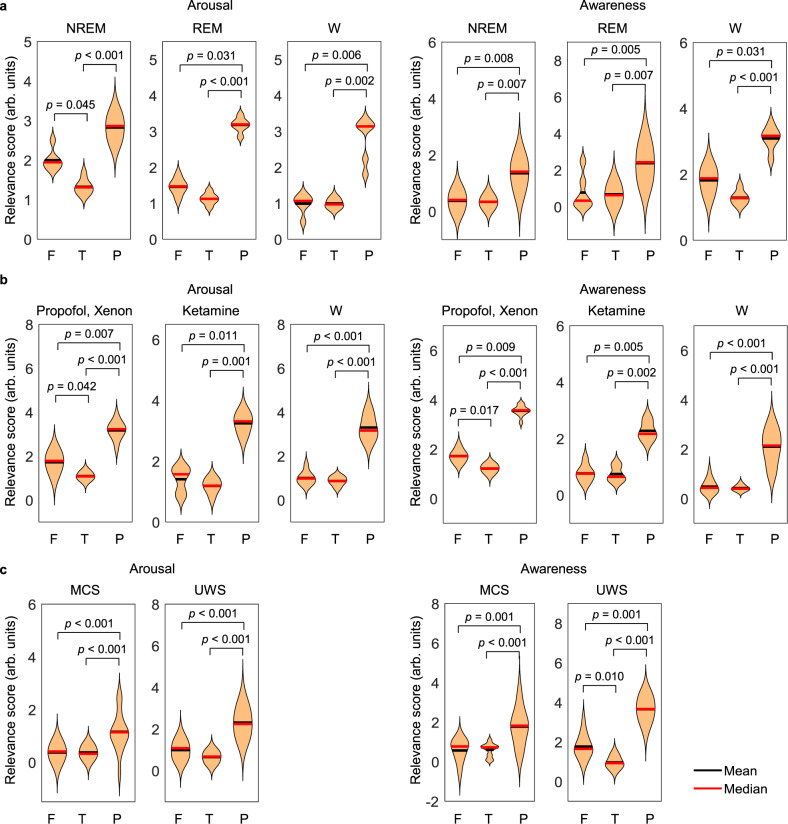
Relevance scores from LRP in TMS–EEG. **a** Sleep and normal wakefulness, **b** anesthesia and wake, and **c** patients with DoC. The violin plots depict the average relevance scores over the frontal, temporal, and parietal regions in all participants. The exact *p*-value corresponding to the significance level was shown using two-sided multiple *t*-tests with Fisher’s least significant differences method for multiple comparisons. [arb. units] denotes an arbitrary unit. F frontal region, T temporal region, P parietal region.

We further checked what the classifier learned through CNN and how it was able to derive those results using LRP. This algorithm describes the predictions of CNN in a given dataset using relevance scores^30^. Figure 7 shows the relevance scores for arousal and awareness among the frontal, temporal, and parietal regions at the scalp level for calculating ECI using TMS–EEG. A high relevance score implies that the trained model recognized the brain region that determines whether it is low or high arousal and awareness. Specifically, brain regions with higher relevance scores indicate that brain signals over that region contributed more to the decision of the classifier on whether arousal and awareness were low or high. In the three conditions (sleep, anesthesia, and patients with DoC), the relevance score over the parietal region was higher than those in the frontal and temporal regions at the group level for both arousal and awareness. The detailed statistical results are reported in Supplementary Table 5. However, since most TMS sites targeted the parietal cortex, it can be argued that the relevance of parietal regions to correctly classify datasets may be biased. Therefore, we investigated the relevance scores with only non-parietal stimulations in patients with DoC. As a result, the parietal region had statistically higher relevance scores than the frontal and temporal regions in both arousal and awareness (Supplementary Fig. 11 and Table 6). Similarly, there was a higher relevance score over the parietal region in both arousal and awareness when calculating ECI from resting-state EEG (Supplementary Fig. 12 and Table 7).

We additionally compared the classification performance of ECI^awa^ among patients with DoC using TMS–EEG data when we excluded electrodes in different brain regions from the input during classification. Consequently, AUC was 1.0 when using all electrodes; however, AUC values were 0.867 and 0.680 when removing frontal and parietal electrodes, respectively (Supplementary Fig. 13).

## Discussion

We show that ECI clearly distinguishes between low and high states of arousal and awareness in TMS–EEG results under sleep, anesthesia, and patients with DoC. Our results suggest that this proposed indicator could similarly be used for resting-state EEG data without TMS under anesthesia and patients with DoC, yielding the same degree of accuracy. In addition, a high correlation with PCI, which measures the integrated EEG response of the thalamocortical system to a direct perturbation induced by TMS^16^, proves that ECI^awa^ is reliable using TMS–EEG data. It also shows that the two measures calculated entirely independently using different methods resulted in the same conclusion, which is a sign that deep learning is indeed a valid and reliable approach. For an ML-based indicator, ECI can be calculated using very few trials. Furthermore, because the classifier learned specific features of the data on its own, our indicator can be computed regardless of whether TMS is applied or its location. Therefore, ECI is a significantly practical and reliable indicator to evaluate levels of consciousness under various conditions. Our analyses using LRP highlighted the major role of the parietal region in determining consciousness, as the classifier primarily uses brain activity in this lobe for predicting low and high states of arousal and awareness.

TMS–EEG responses under sleep exhibited well-known phenomena^31^. In wakefulness, TMS generates a series of low-amplitude high-frequency activities related to cortical flow in long-range connections^32^. A similar long-lasting response is evoked during REM sleep with subjective experience^33^. During NREM sleep with no subjective experience, TMS triggers larger, low-frequency activity that quickly dissipates^32^, which is the hallmark of bistability in the thalamocortical system. Cortical effective connectivity is also broken down during NREM sleep^33,34^. The brain response to TMS perturbation was already used to distinguish the levels of awareness, irrespective of sensory processing and motor responses under physiological, pharmacological, and pathological conditions^34–36^. Therefore, this TMS-evoked response was applied to our end-to-end CNN framework. Similar to several studies using ML, we observed higher two-class (low or high) classification accuracy using CNN when compared to linear discriminant analysis (LDA) and support vector machine (SVM) for both arousal and awareness. This suggests that our framework is especially relevant for EEG results, which possess several nonlinear features. The classification performance of spatiotemporal information was higher than that of spatio-spectral information. Using our framework, it was shown that temporal information discriminates different levels of consciousness more clearly than spectral information, as the functional connectivity associated with consciousness changes in both space and time^37^. However, this does not imply that temporal information is more important than spectral information for distinguishing consciousness. Temporal information has more distinct characteristics than spectral information for predicting the state of consciousness in the proposed framework. Nevertheless, PCI also used spatiotemporal dynamics in TMS-evoked responses^16^, which is significantly important for distinguishing consciousness.

We applied transfer learning to a single domain as well as multiple domains. In the sleep domain, classification performance was high when trained on only the sleep domain or together with the anesthesia domain. Due to a large number of trials in the sleep domain, training was performed satisfactorily using only sleep data. The distance of the averaged TMS-evoked potentials under the domains of anesthesia and sleep was significantly close, which indicated that the two domains were highly similar^38^. Thus, a close distance implies that the two domains have similar patterns, and the classification performance indeed increased when these domains were trained together. In the anesthesia and DoC domains, the trials of a single domain were not sufficient; thus a higher classification performance was achieved when trained with similar domains. In addition, brain signals change over time, even when recorded with the same participants because of physiological and psychological differences over time^27^. Therefore, participant-independent learning such as the LOPO cross-validation is considerably difficult, as opposed to participant-dependent learning^24^. We solved these problems via transfer learning utilizing multiple domains and participant-independent ECI.

ECI, by averaging interclass probability, distinguished whether each state was low or high in both arousal and awareness. Because of its inherently high two-class performance, it was possible to calculate the discriminable ECI using a few trials in a single session. The criteria used for determining ECI^aro^ and ECI^awa^ were different, especially for REM sleep, ketamine-induced anesthesia, and UWS patients. Although the dataset was the same, these states were trained by different labels depending on arousal and awareness. For instance, REM sleep is high in awareness but low in arousal. The proposed classifier learns models for these different criteria by training itself based on the criteria of arousal and awareness. Specifically, because the learning ability of the CNN is derived from the automatic extraction of complicated representations from EEG signals^39^, it can properly distinguish between both states, even if the same state has different labels depending on the criteria. We further used data from MCS* patients as verification samples. MCS* patients were correctly predicted, since data from UWS and MCS patients were included during training, and the classifier is independent of behavior.

According to the LRP, we observed higher relevance scores in the parietal regions, compared to other regions at the scalp level. This was observed for all data, including TMS–EEG and resting-state EEG results. The brain regions that led to decision-making were similar in arousal and awareness. However, this does not imply that arousal and awareness are supported by the same underlying neurophysiological mechanisms. The relevance scores simply explain the patterns of cortical EEG activity resulting in this classification.

In sleep and healthy wakefulness, when arousal and awareness were high, they were highly relevant in the parietal region. This EEG feature, which distinguishes high and low states in arousal and awareness, can be interpreted in line with the posterior hot zone of consciousness^40^. Local changes in this parietal region are associated with the occurrence of dreaming and unconscious sleep^41,42^, and our framework may learn from EEG pivotal features recorded in this area. The importance of the posterior hot zone has already been emphasized using the within-state sleep paradigm^43,44^. Similar to sleep, we observed high relevance scores for the parietal region in the domains of both anesthesia and patients with DoC. This implies that EEG activity in this region had a decisive effect in determining the high and low states of arousal and awareness. The increased slow-wave activity was observed under propofol- and xenon-induced anesthesia when compared to healthy wakefulness before drug administration^36^. In addition, just as cortical neurons induced bistable changes during NREM sleep, TMS in propofol-mediated anesthesia-induced low-amplitude, low-frequency, positive–negative potentials, and TMS in xenon-mediated anesthesia caused a significantly large amplitude but stereotyped positive–negative deflection^36^. Moreover, under ketamine-induced anesthesia, TMS-evoked response was determined to be similar to REM sleep, which features dreaming during a low state of arousal^36^. Previous studies have shown that the change in slow waves induced by propofol is primarily observed in the posterior hot zone^45^ and the posterior main hub is disrupted during anesthesia-induced alteration of consciousness^7^. In UWS patients, TMS triggered a local and slow response similar to NREM sleep and general anesthesia, whereas MCS patients showed complex TMS-evoked responses^33^. Similarly, differences in alpha connectivity between the UWS and MCS patients are apparent within the posterior hot zone^46^. That is, when determining whether arousal and awareness are low or high, our classifiers used differences and changes in EEG activity over the parietal region to make decisions. Particularly, similar results were observed in patients with DoC using several TMS target sites, and in sleep and anesthesia domains, where the parietal region was primarily stimulated. This suggests that our findings regarding the parietal region are unrelated to the TMS target site. Thus, our trained model used neurophysiological features to classify whether arousal and awareness are low or high. This indicates an appropriate design of our model as the classification decision was primarily based on EEG signals over the parietal region, which is suggested to be a hot spot of consciousness, compared to the frontal region^43^. The difference in the parietal region could be clearly identified through correct trials during sleep and healthy wakefulness. It is meaningful that the frontal region contributed less than parietal regions in the context of the controversy regarding the spatial localization of the neural correlates of consciousness^40,45^. Considering the subcortical influences related to striatal-thalamic circuits, it has been recently observed that the parietal region contributes more to the levels of consciousness than the frontal region^47^. The implication of the parietal cortex in consciousness has also been demonstrated in other neuroimaging modalities, such as functional magnetic resonance imaging^48^ and magnetoencephalography^49^.

This study does have certain limitations. First, our sample size was relatively small. In the future, it will be necessary to further test the reliability of the proposed indicator with larger cohorts and validate it at the clinical level before implementing it in a clinical setting. Sleep experiments would also have to be applied to more participants in the future. Second, we explored the possibility of calculating ECI with a minimal amount of data, up to a single trial. However, we did not attempt to measure ECI in real time. Thus, in the future, ECI could be calculated in real-time for practical application. Third, ECI does not differentiate between physiological, pharmacological, and pathological conditions, but distinguishes between high and low states of arousal and awareness. ECI can thus distinguish between REM sleep (or ketamine) and wakefulness. It may also be difficult to select the model to use when calculating ECI since the domain has to be known beforehand. Nevertheless, if a single domain has sufficient trials, the LOPO approach would be the most accurate. Another limitation might be related to the possible contamination of TMS-EEG data by auditory and somatosensory components. As in previous studies^17,35,36^, to avoid auditory and somatosensory co-stimulation, participants wore earphones with noise masking and a thin foam between the scalp and the TMS coil that was used. Although it is difficult to systematically rule out the contribution of sensory co-stimulation in every measurement, the application of effective noise-masking procedures^50^ and the real-time monitoring of data quality during the acquisition^51^ may significantly mitigate this issue^52^. Finally, ECI indicates whether arousal and awareness are low or high and cannot be considered functional. It, therefore, should be developed into a functional index.

In conclusion, we proposed ECI as a neurophysiological indicator to simultaneously discriminate the levels of arousal and awareness in modified states of consciousness. This tool allows disentangling the levels of consciousness, with a single measure, in different clinical settings such as monitoring surgical interventions (i.e., anesthesia-induced states) and diagnosing patients with DoC. This indicator was validated under different physiological, pharmacological, and pathological conditions, and it reliably disentangled low levels from high levels of both arousal and awareness. Besides, the proposed ECI is considerably accessible and practical, as it can be applied to resting-state EEG without TMS, and requires fewer trials. Therefore, the proposed indicator can be a reliable discriminator and valuable tool as an objective measure of consciousness. As parietal regions appear to be the most relevant for classification, an EEG configuration around that area could be sufficient if ECI is used in clinical practice. These findings could be useful in diagnosing severely brain-injured patients and monitoring their levels of consciousness in real-time, especially in clinical settings where time constraints preclude long-duration assessment. The proposed reliable ECI can provide insights into the classification of conscious levels using deep learning and neural correlates of consciousness.

## Methods

### Datasets

The sleep dataset included six healthy participants (five males, aged 23.7 ± 3.2 years), as previously reported by Nieminen et al.^43^ for NREM data and Lee et al.^53^ for REM data. The inclusion criteria included (i) between 18 and 75 years of age and (ii) in good general health. The exclusion criteria were as follows: (i) neurological, psychiatric, mood, and sleep disorders, (ii) contraindications for TMS (e.g., history of seizures), and (iii) psychotropic medication. All participants provided written consent, and the experimental paradigm was approved by the Institutional Review Board (IRB) at the University of Wisconsin–Madison (HSC-2013-0019). Sleep stages were manually scored every 30 s following the American Academy of Sleep Medicine Scoring Manual. After 3 min or more, when the participant entered a specific sleep stage, TMS was applied over the parietal cortex using a navigated brain stimulation system (eXimia Navigated Brain Stimulation, Nexstim Plc, Finland). Supplementary Table 8 lists the TMS target site and the number of sessions and trials. The participants were awoken by an alarm sound that lasted for 1.5 s after each session. They were then asked if they had had conscious experience. The TMS–EEG experiments were performed over a period of four or five nights per participant.

The anesthesia data were previously published by Sarasso et al.^36^. Sixteen healthy participants (eight males, aged 18–28 years) were included under ketamine- (*n* = 6), propofol- (*n* = 5), and xenon-induced (*n* = 5) anesthesia. The inclusion criteria included (i) older than 18 years and (ii) stability of vital parameters. The exclusion criteria were as follows: (i) neurological, cardiovascular, psychiatric, and mood disorders, (ii) contraindications for TMS (e.g., history of seizures, metal implants such as a pacemaker), and (iii) medical conditions that were incompatible with the anesthesia and/or the TMS procedure. This experimental protocol was approved by IRB at the University of Liège (2009/153 (ketamine), 2007/191 (propofol), and 2009/242 (xenon)); moreover, all participants provided written informed consent. TMS was applied over the left parietal or motor regions after participants reached deep unresponsiveness (a score equal to 5 in the Ramsay scale, which corresponds to no response to external stimuli) following standard anesthetic procedures^36^. The stimuli target site and the number of trials are listed in Supplementary Table 9. In addition, upon waking up from anesthesia, reports about the conscious experience during anesthesia were collected. Conclusively, the participants reported little conscious experience during propofol- and xenon-induced anesthesia, but vivid dreams were experienced during ketamine-induced anesthesia.

For patients with severe brain injury, the data of six UWS patients (2 males, 4 traumatic brain injuries, time since the injury of 10.6 months (1–47), age: 36.2 ± 28.6 years) and ten MCS patients (7 males, 5 traumatic brain injuries, time since the injury of 65.7 months (1–343), age: 44.6 ± 20.5 years) were previously reported by Bodart et al.^54,55^ and Rosanova et al.^56^. This study was approved by the Ethics Committee of the Medicine Faculty of the University of Liège (ref 2009/52) and written informed consent was obtained from legal representatives of all patients. All of them fell into a coma due to brain injury and presented a prolonged state of impaired consciousness. The inclusion criteria included (i) older than 18 years and (ii) diagnosis of DoC following a severe acquired brain injury. The exclusion criteria for patients were as follows: (i) patients having significant neurological, neurosurgical, or psychiatric disorders prior to the brain injury that leads to DoC, (ii) patients having any contraindication to TMS–EEG or magnetic resonance imaging (electronic implanted devices, active epilepsy, external ventricular drain), and (iii) patients who were not medically stable. Accredited experts performed repeated CRS-R for each patient, including on the day of the TMS–EEG examination and before the fluorodeoxyglucose-positron emission tomography (FDG-PET) scan. The FDG-PET is a reliable and sensitive tool to detect MCS* patients based on the previous literature^15^. MCS* patients were the patients who were diagnosed with a UWS with the CRS-R at the bedside but diagnosed as an MCS based on the FDG-PET data (that is, patients showing relative metabolic preservation of the frontoparietal network based on a subjective visual assessment of the Statistical Parametric Mapping analysis^15^). TMS–EEG data were acquired similar to previous studies^16,35^. Moreover, added data were newly included as follows: 9 UWS patients (6 males, 5 traumatic brain injuries, time since the injury of 6.2 months (1–13), age: 41.4 ± 21.1 years), 5 MCS patients (4 males, 2 traumatic brain injuries, time since the injury of 63.0 months (2–169), age: 32.4 ± 14.0 years), and 4 MCS* patients (2 males, 3 traumatic brain injuries, time since injury 18.5 months (3–52), age: 36.3 ± 10.5 years). This study was approved by the Ethics Committee of the Medicine Faculty of the University of Liège (ref 2012/55) and all legal representatives of patients provided written informed consent before the experiments; moreover, newly recorded data used exactly the same procedure as before. These added data were recently acquired by Dr. Olivia Gosseries & Pr. Steven Laureys team at the University of Liège. For the added data, the inclusion and exclusion criteria for patients were exactly the same as the previously used data. These data are part of a bigger study conducted in the frame of the Human Brain Project. The final dataset consisted of 15 UWS patients, 15 MCS patients, and 4 MCS* patients. The detailed demographic and clinical information of severely brain-injured patients is listed in Supplementary Table 10. The TMS target site was selected using a neuronavigation system over the parietal, motor, or premotor regions, avoiding structural lesions using the magnetic resonance imaging data of the patient. The stimuli target sites for all participants are listed in Supplementary Table 11. The participants remained awake or were kept awake using the CRS-R arousal protocol in between TMS stimulation^12^.

For the four datasets, EEG data were recorded using a 60-channel TMS-compatible amplifier and a two-channel electrooculogram (Nexstim eXimia, Nexstim Plc, Finland) with a 1450 Hz sampling rate. During all the sessions, earphones presenting white noise were used to reduce the noise of the TMS pulses and we used a thin foam between the scalp and the TMS coil to avoid somatosensory evoked potentials. These pulses were presented at random intervals of 2–2.3 s using a figure-of-eight coil. The maximum electric field was between 100 and 130 V/m at the TMS target site. In particular, the TMS stimulation was always performed in the medial half of one hemisphere, to avoid any muscle artifacts.

Finally, we used resting-state EEG without TMS for the domains of anesthesia and severely brain-injured patients. These data were acquired using the same participants as in the TMS–EEG experiments. We used the ketamine- (*n* = 5), propofol- (*n* = 5), and xenon-induced anesthesia data (*n* = 5) as previously reported by Sarasso et al.^36^. For at least 3 min before the TMS–EEG experiments, resting-state EEG data were recorded during anesthesia and each state of wakefulness before the anesthesia. With regard to the severely brain-injured patients, 5-min resting-state EEG for MCS patients (*n* = 15) and UWS patients (*n* = 15) was included. In addition, four MCS* patients were added along with the same participants for whom TMS–EEG was recorded. The sampling rate was 1450 Hz. Finally, among previously published data, the data with a signal-to-noise of 1.4 or less were excluded from the analysis^16^.

### Data preprocessing

TMS–EEG data were preprocessed using the SiSyPhus Project MATLAB program (University of Milan, Italy) and the EEGLAB toolbox^57^. The signals were down-sampled to 362.5 Hz and band-pass filtered between 0.5 and 45 Hz using a second-order Butterworth filter. The signals of −400 to 1000 ms were segmented and baseline-corrected using the 400 ms baseline before the TMS pulses. Bad channels were manually detected and interpolated using superfast spherical interpolation for artifact removal. We also discarded the components related to eye movements using independent component analysis and removed trials setting a threshold of ±100 μv affected by ocular artifacts, other artifacts, or noise. The data were re-referenced to an average reference^58,59^.

Resting-state EEG data were processed using the EEGLAB toolbox^57^. Preprocessing was performed using a process similar to that used with TMS–EEG data, with segmentation being performed every 1 s. The number of trials for each session we used is listed for patients under anesthesia (Supplementary Table 12) and severely brain-injured patients (Supplementary Table 13).

### Proposed framework for calculating an ECI

Step 1—Extraction of EEG features: In all trials of all TMS–EEG data, we used the 200–400 ms time window of data after the TMS regardless of the lateralization or target site of the TMS in sleep, anesthesia, and for patients with severe brain injury. More details related to this deliberate choice are reported in Supplementary Note 2. In resting-state EEG, only the first 200 ms of data were used from the segmented 1 s of data. In the first step, EEG data were converted from 2D raw signals to 3D input. To preserve the spatial information and characteristics of EEG, we used spatio-spectral and spatiotemporal 3D features. The raw EEG signals at time index *t* are measured in a 1D data vector ${r}_{t}={\left[{s}_{t}^{1},{s}_{t}^{2},{s}_{t}^{i},\cdots ,{s}_{t}^{n}\right]}^{T}$, where ${s}_{t}^{i}$ is the acquisition data by the $i$th electrode channel at timestamp *t*. *n* indicates the number of electrode channels. However, these simple signals do not capture all the spatial information characteristics in the brain. Therefore, we converted 1D data vectors to 2D EEG data meshes using the spatial information of the electrode location. Zero was inserted in the place of a *null* electrode in 2D matrices at time index *t*^60^. Finally, we calculated 3D data by adding spectral or temporal information (Supplementary Fig. 14). Specifically, spectral information was divided into 5 frequency bands: delta (1.5–4 Hz), theta (4–8 Hz), alpha (8–13 Hz), beta (13–30 Hz), and gamma bands (30–40 Hz)^44^. Finally, a 10 × 11 × 5 matrix using spectral information and a 10 × 11 × 72 matrix using temporal information were used as the final CNN inputs (here, 10 × 11: the converted matrix of spatial information; 5: delta, theta, alpha, beta, and gamma bands of spectral information; 72: 200–400 ms of temporal information).

Step 2—Calculation of interclass probability using CNN: The model was trained to distinguish both arousal and awareness in terms of whether they were low or high. We first calculated the domain similarity based on the cosine distance for domain transfer learning. The cosine distance *D* between domains *A* and *B* is defined as follows:

1 $$D\left(A,\,B\right)=1-\,\frac{{f}_{A}^{{{{\rm T}}}}{f}_{B}}{\parallel {f}_{A}\parallel \parallel {f}_{B}\parallel}$$

where $\parallel \bullet \parallel$ indicates the norm of a vector. This similarity was used when selecting the source domain for domain transfer learning^38^. In calculating the similarity according to the states of arousal and awareness, the labels in each state are different. During sleep, NREM sleep with no subjective experience and REM sleep with subjective experience were learned as low arousal, whereas healthy wakefulness was learned as high arousal. Conversely, in awareness, REM sleep with subjective experience and healthy wakefulness indicated by open eyes were learned as high awareness, while NREM sleep with no subjective experience was learned as low awareness. Specifically, we used stage 3 of NREM sleep for clear feature extraction of deep sleep. Under general anesthesia and in healthy wakefulness before anesthesia, the arousal state was divided as follows: (i) low, when under ketamine, propofol, and xenon-induced anesthesia, and (ii) high, when in healthy wakefulness before anesthesia. Conversely, the awareness state was divided into low (under propofol and xenon-induced anesthesia) and high (under ketamine-induced anesthesia and healthy wakefulness before anesthesia). Finally, in patients with DoC, UWS and MCS patients were distinguished in terms of awareness: low (UWS patients) and high (MCS patients). The UWS and MCS patients corresponded to high arousal.

Deep learning was conducted in a MATLAB environment powered by a TITAN V GPU. We used the LRP toolbox^30^ for CNN classification and interpretation. The CNN was applied to the two components of consciousness (arousal and awareness). In each architecture, we inserted five convolutional layers with 2D filters for the deep neural network. The first layer with 100 filters and the second layer with 80 filters featured kernel sizes of 3 × 3 and 2 × 2 with stride 1 × 1, respectively. Then, a max-pooling layer with a pool size of 2 × 2 and stride 1 × 2 was added. Similarly, two convolutional layers with kernel sizes of 3 × 3 (with stride 1 × 1) and 2 × 2 (with stride 2 × 1) were subsequently used. After max-pooling with a pool size of 2 × 1 and stride 2 × 1, a final convolutional layer comprising two filters with a kernel size of 1 × 1 and stride 1 × 1 was incorporated. Finally, the generated feature maps were flattened into a 1D vector. A softmax layer was used for classification. In the softmax layer, each element indicates the probability that the original input belongs to the corresponding class. In this training procedure, the parameters in the deep neural network were learned through back-propagation. The activation function in each convolutional layer was a rectified linear unit. The detailed CNN architecture is presented in Supplementary Table 14. The Adam optimizer was used with an initial learning rate of 0.005, and the learning rate was updated to sublinear for learning rate decay during an evaluation step of training. Specifically, we used hyperparameters as values of 0.9 for β1, 0.999 for β2 for Adam optimizer^61^. The batch size was 25 for training, and the maximum number of training iterations was five times the number of training data. Consequently, the output of this architecture was the probability of each class.

For a comparison with other classifiers, we also considered an LDA classifier^62^ and SVM with polynomial kernel function^63^ using the same input data as fair baseline methods. The classification performance was measured in LOPO-nested cross-validation for the generalized neural network. This method is a special case of *k*-fold cross-validation. Specifically, all participants except one (the target participant) were used for training, and the target participant was then tested using the classifier. In the training phase, 80% of the datasets were used to learn the classifier, and the remaining 20% were reserved for validation. This process was repeated for each participant. Further, internal validation sets (inner cross-validation) were performed to choose the hyperparameters of the model^64^. Thus, the same hyperparameters were selected in the external validation sets^65^. The LOPO cross-validation procedure uses data efficiently and can reduce overfitting. It also provides unbiased estimates of the averaged classification error for all possible training sets^66^. The same LOPO-nested cross-validation is applied in LDA and SVM using the Berlin brain–computer interface toolbox^67^. Three possible values (0.0001, 0.01, and 1) were chosen as penalties for misclassification in the SVM model^65^. For spatio-spectral input and spatiotemporal input 1 and 0.0001 were used respectively.

Step 3—Calculation of ECI in a single session: We obtained each interclass probability according to two components (arousal and awareness) at the previous step. In each TMS session, the interclass probability was averaged to calculate an ECI. This approach has the advantage of being able to offset outliers.

2 $${C}_{j}=\,\frac{1}{N}\mathop{\sum }\limits_{i=1}^{N}{p}_{i}$$

Here, ${p}_{i}$ is the probability of high arousal or high awareness from each trial $i$ among the probability values (high versus low) for the two classes from the softmax function in the CNN. *N* is the number of trials in a single session *j*. The averaged interclass probability ${C}_{j}$ from arousal is the value of the *x*-axis as ECI^aro^, and the averaged interclass probability ${C}_{j}$ from awareness becomes the value of the *y*-axis as ECI^awa^. Consequently, ECI is expressed as a 2D indicator representing both arousal and awareness simultaneously. As mentioned earlier, we used the LOPO cross-validation. It is to be noted that only data from one participant were used as the test, and the data from the remaining participants were used for training. Finally, the test and training data did not demonstrate an overlap at all.

Step 4—Interpretation using LRP: We used LRP based on a backward-propagation mechanism for interpretability of the deep neural networks. This calculated the pixel-based decomposition process^28^.

3 $$\mathop{\sum }\limits_{p=1}^{d}{R}_{p}=f({{{{{\rm{x}}}}}})$$

Here, x = (${x}_{1},\ldots {x}_{d}$) indicates an input vector and $f({{{{{\rm{x}}}}}})$ is the model output. The relevance score ${R}_{p}$ is the decomposition of the prediction for the input ${x}_{p}$. This score is calculated through the backward propagation of the model input. Therefore, relevance scores describe a single nonlinear decision for the output corresponding to each input^68^. Through this method, we can observe not only the interpretation of classification decisions but also what features the model has learned^28^. To investigate which brain regions and signals caused these classification results, we compared the relevance scores from the LRP by dividing them into the following three regions^69^: the frontal (Fp1–2, Fpz, AF1–2, AFz, F1–8, and Fz), temporal (FT9–10, T7–8, TP9–10), and parietal (CP1–6, CPz, P1–4, P7–8, and Pz) regions. We focused on the frontal, temporal, and parietal regions when comparing the relevance score resulting from the LRP as there is an ongoing debate regarding which brain area, i.e., the front versus the back, is related to consciousness^40^. We also included the temporal region as activation in the NREM sleep increases in this region^70^.

Additional EEG analyses are shown in Supplementary Methods as follows: (i) performance according to the number of trials in the ECI calculation, (ii) comparison of the difference between correct and incorrect trials, and (iii) classification performance using EEG signals excluding frontal or parietal electrodes.

### Comparison with PCI

We compared ECI^awa^ with the PCI values computed following the same procedure described in^16^. PCI measures the complexity of the spatiotemporal patterns of cortical activity significantly evoked by TMS^16^. PCI ranges between 0 (minimum complexity) and 1 (maximum complexity). Previous extensive validation of PCI provided an empirical cutoff (PCI* = 0.31) to discriminate between consciousness and unconsciousness^17^.

### Statistical analysis

We used the Kruskal–Wallis test (nonparametric one-way analysis of variance) to analyze the differences in the classification accuracy; moreover, two-sided multiple *t*-tests were used for post-hoc analysis using Fisher’s least significant differences method for multiple comparisons to compare the classification performance of the three classifiers (LDA, SVM, and CNN) of sleep data for each component (arousal and awareness) and at three-time ranges (0–200, 200–400, 400–600 ms) of spatiotemporal information. The Kruskal–Wallis test was also performed to compare the classification performance using transfer learning. Similarly, Fisher’s least significant differences method was applied after the two-sided multiple *t*-tests.

To investigate the discrimination of ECI in each state of consciousness, the feedforward network was trained with 20 hidden layers using the LOPO approach. For each output class, the AUC, sensitivity, and specificity were calculated using ROC analysis.

The Kruskal–Wallis test was employed to investigate if there were any differences in relevance scores among brain regions from LRP under sleep. We performed two-sided multiple *t*-tests using Fisher’s least significant differences method for multiple comparisons. In addition, the Kruskal–Wallis test was performed to explore the differences in relevance scores from LRP under the condition of anesthesia and for severely brain-injured patients. For post-hoc analysis, two-sided multiple *t*-tests were performed using Fisher’s least significant differences method for multiple comparisons.

Finally, we used Pearson’s correlation to investigate the relationship between ECI^awa^ and PCI. In this study, all significances were indicated by ${{{{{\rm{\alpha }}}}}}$ = 0.05.

### Reporting summary

Further information on research design is available in the Nature Research Reporting Summary linked to this article.

## Supplementary information

Supplementary Information

Reporting summary

## Data Availability

All data (sleep dataset, anesthesia dataset, already published brain injury dataset, and new added brain injury dataset) generated and used in this study have been deposited in a local database and are available upon reasonable request to Olivia Gosseries. In addition, resting-state EEG signals during anesthesia and wake are available online upon request at the repository Zenodo (10.5281/zenodo.806176). The TMS–EEG data of some brain injury patients (published and new datasets) will also be freely available through EBRAINS within 2022 with no restriction to access (10.25493/G8E3-DQE). The sleep dataset was previously reported by Nieminen et al.^43^ for NREM data and Lee et al.^53^ for REM data. The anesthesia data were also previously published by Sarasso et al.^36^. The published brain injury dataset was previously reported by Bodart et al.^54,55^ and Rosanova et al.^56^. However, new added brain injury dataset has not yet been published. The raw EEG data are protected and are not made publically available owing to data privacy laws, but are available from the corresponding author upon reasonable request. Source data are provided with this paper.

## Source data

Source Data

## Code availability

Source code generated and used for this study is publicly available for download at https://github.com/MinjiLee-ku/ECI and 10.5281/zenodo.5760787 (ref.^71^). Source code for CNN and LRP is freely available at https://github.com/sebastian-lapuschkin/lrp_toolbox. Source code for violin plot is available from https://www.mathworks.com/matlabcentral/fileexchange/45134-violin-plot. Source code for shaded error bar is available from https://github.com/raacampbell/shadedErrorBar.
