# Supplementary material for: Quantifying arousal and awareness in altered states of consciousness using interpretable deep learning
Source: Nat Commun. 2022 Feb 25;13:1064. doi: 10.1038/s41467-022-28451-0 (PMC8881479; doi:10.1038/s41467-022-28451-0)
Supplement: Supplementary file 1 — Supplementary Information [file 41467_2022_28451_MOESM1_ESM.pdf]

Supplementary information

Quantifying arousal and awareness in altered states of consciousness using interpretable deep learning

Minji Lee[1], Leandro R. D. Sanz[2,3], Alice Barra[2,3], Audrey Wolff[2,3], Jaakko O. Nieminen[4,5], Melanie Boly[4,6], Mario Rosanova[7,8], Silvia Casarotto[7,9], Olivier Bodart[2], Jitka Annen[2,3], Aurore Thibaut[2,3], Rajanikant Panda[2,3], Vincent Bonhomme[10,11,12], Marcello Massimini[7,9], Giulio Tononi[4], Steven Laureys[2,3], Olivia Gosseries[2,3,4,13,*], and Seong-Whan Lee[14,*]

[1]Department of Brain and Cognitive Engineering, Korea University, Seoul, Republic of Korea

[2]Coma Science Group, GIGA-Consciousness, GIGA research center, University of Liège, Belgium

[3]Centre du Cerveau[2], University Hospital of Liège, Liège, Belgium

[4]Wisconsin Institute for Sleep and Consciousness, Department of Psychiatry, University of Wisconsin, Madison, USA

[5]Department of Neuroscience and Biomedical Engineering, Aalto University School of Science, Espoo, Finland

[6]Department of Neurology, University of Wisconsin, Madison, WI, USA

[7]Department of Biomedical and Clinical Sciences “L. Sacco”, University of Milan, Milan, Italy

[8]Fondazione Europea di Ricerca Biomedica, FERB Onlus, Milan, Italy

[9]IRCCS Fondazione Don Carlo Gnocchi ONLUS, Milan, Italy

[10]Department of Anesthesia and Intensive Care Medicine, University Hospital of Liège, Liège, Belgium

[11]University Department of Anesthesia and Intensive Care Medicine, CHR Citadelle, Liège, Belgium

[12]Anesthesia and Intensive Care Laboratory, GIGA-Consciousness, GIGA research center, University of Liège, Liège, Belgium

[13]Department of Psychology, University of Wisconsin, Madison, USA

[14]Department of Artificial Intelligence, Korea University, Seoul, Republic of Korea

* Co-corresponding author: O. Gosseries (ogosseries@uliege.be) and S.-W. Lee (sw.lee@korea.ac.kr)

Supplementary Note 1: TMS-evoked responses according to TMS target sites

In our study, the sleep and anesthesia-based experiments targeted the parietal region (excluding one participant who underwent xenon-induced anesthesia, which was stimulated on the motor cortex). In the case of patients, we stimulated the parietal and premotor regions; however, there was no response at certain times; therefore, we recorded TMS–EEG data at different locations. In our data, the TMS target sites in the UWS and MCS patients were over the premotor, motor, and parietal regions. Therefore, to overcome variability due to different TMS target sites, we observed TMS-evoked response in patients with UWS when several TMS target sites were applied. Supplementary Figure S1a shows the TMS-evoked potentials at four TMS target sites (right premotor, left motor, left parietal, and right parietal regions) in four different UWS patients. As it can be observed at 15–100 ms, there were high potentials at the TMS target site. Earlier TMS-evoked potentials were more local and dependent on the TMS target site than later TMS-evoked components. However, at 200–400 ms, the effects on the TMS target site disappeared. We additionally compared the TMS-evoked responses when stimulating left or right parietal regions during NREM sleep with no subjective experience (Supplementary Figure S1b). Similarly to UWS patients, initial TMS-evoked responses were influenced by the TMS target site while later responses were less impacted. The spatiotemporal profile at 200–400 ms were almost similar when stimulating left or right parietal regions. Therefore, we used the results after 200–400 ms post application of TMS for the calculation of ECI.

Supplementary Note 2: Comparison of single-trial classification performance according to input types and classifiers

We first focused on TMS–EEG data collected during both sleep and wakefulness. During NREM sleep, TMS yielded a local and stereotypical positive–negative response, similar to the spontaneous oscillations of sleep slow waves. In contrast, during REM sleep and healthy wakefulness, TMS triggered a rapidly changing and spatially differentiated cortical response (Supplementary Figure S2).

Two types of information were considered for the type of 3D input of the classifier, which are as follows: (i) data representation 1 (DR1): spatio-spectral information and (ii) data representation 2 (DR2): spatiotemporal information. Under sleep and healthy wakefulness, we classified TMS–EEG data (200–400 ms after TMS) using a CNN based on low or high states of arousal and awareness. LDA and SVM were applied as comparative classifiers. In the LOPO cross-validation, the data of five out of six participants, excluding one participant (target) to be tested, were trained. Then, the data of the excluded target participant were tested. Because there were six participants in the sleep dataset, this process was repeated six times (Supplementary Figure S3a). No information on the target participant was included in the training phase. The classification performance for arousal and awareness in the sleep experiment is listed in Supplementary Table S1. The classification accuracy differed depending on the classifier (LDA, SVM, and CNN) and the input type (DR1 and DR2) for both arousal ($Chi\text{-}square_{(5, 30)} = 19.49$, $p = 0.002$) and awareness ($Chi\text{-}square_{(5, 30)} = 21.82$, $p < 0.001$). The highest classification accuracy was 87.79 ± 2.74% (mean ± standard deviation) and 91.95 ± 5.19% using CNN based on DR2 for the arousal and awareness states, respectively (Supplementary Table S2). Excluding SVM-DR2, the two-class classification accuracy of CNN-DR2 was significantly higher than that of the other classifiers and inputs. Further, although there were no statistical differences between CNN-DR2 and SVM-DR2 after multiple comparison corrections, the performance of CNN-DR2 was superior to that of SVM-DR2 for all six participants. Consequently, the classification performance using CNN based on DR2 was higher than other inputs (i.e., DR1) and classifiers (i.e., LDA and SVM). Therefore, in all subsequent classifications, CNN-DR2 based on the LOPO approach was used.

We speculate that the data within the time interval 200–400 ms best discriminate between consciousness and unconsciousness in DoC patients ($Chi\text{-}square_{(2)} = 14.71$, $p < 0.001$; see Supplementary Table S3) because they are less affected by the TMS stimulation site. Indeed, the classification accuracy was 55.79 ± 24.23%, 75.84 ± 14.71%, and 57.71 ± 20.36% for each interval of 200 ms (0–200, 200–400, and 400–600 m) after TMS, respectively. When considering the

classification performance in awareness using the data within the three time periods, accuracy was 91.42 ± 4.79%, 91.95 ± 4.74%, and 90.85 ± 5.11% for sleep and 82.50 ± 12.31%, 80.20 ± 10.06%, and 74.42 ± 20.96% for anesthesia using each interval of 200 ms (0–200, 200–400, and 400–600 m), respectively. There was no significant difference in accuracy for each interval of 200 ms in both sleep and anesthesia (Sleep: $Chi\text{-}square_{(2)} = 0.50$, $p = 0.77$; anesthesia; $Chi\text{-}square_{(2)} = 0.81$, $p = 0.67$). We speculate that uniformly high accuracy in sleep and anesthesia conditions across time intervals is supported by the fact that TMS stimulation sites are consistent across participants and between conditions (wake vs. sleep and wake vs. anesthesia). We believe that, in DoC patients, the time interval 0–200 ms does not provide satisfactory performances possibly because early EEG responses to TMS may significantly differ across stimulation sites at early latencies. Thus, we deliberately selected the second interval (200–400 ms) in order to make the proposed framework universally applicable across conditions (sleep, anesthesia, and DoC) and across different TMS target sites.

Supplementary Note 3: Similarity in TMS-evoked responses using the hierarchical clustering for transfer learning

The averaged TMS-evoked potentials after 200–400 ms were compared to investigate the relationship among the three domains. We calculated the domain similarity distance between sleep, anesthesia, and patients with DoC domains based on cosine distance (Supplementary Figure S4). A small cosine distance between two domains indicates high similarity between these domains. Low arousal states in the sleep (NREM and REM sleep) and anesthesia domains (ketamine, propofol, xenon) were close to each other. High arousal states in the sleep (healthy wakefulness before sleep) and anesthesia domains (wakefulness before anesthesia) were also close and formed a cluster. Similarly, low awareness states in the sleep (NREM) and anesthesia domains (propofol, xenon) were adjacent to each other. Further, high awareness states in the sleep domain (REM and healthy wakefulness) were also close to high awareness states in the anesthesia domain (ketamine and wakefulness before anesthesia). During

arousal, the high state in the patients with DoC domain (MCS and UWS patients) was surprisingly close to the low state, especially in the low state of anesthesia (ketamine, propofol, and xenon).

Supplementary Note 4: Comparison of classification performance for transfer learning

In TMS–EEG data, we classified arousal and awareness states into two classes (low versus high) to use domain transfer learning (Table 2). When the LOPO cross-validation was applied in domain transfer learning, all data, excluding those of one participant, were trained for calculating ECI in the target domain. Thus, if the source domains were sleep (n = 6) and anesthesia (n = 16), and the target domain was sleep, then 21 data points were trained, excluding one of the 22 participants, to calculate ECI. As there were six participants in the sleep dataset, this was repeated six times (Supplementary Figure S3b). For arousal, there were differences in classification performance (Sleep: $Chi\text{-}square_{(3, 20)} = 8.33$, $p = 0.039$; Anesthesia: $Chi\text{-}square_{(3, 60)} = 10.15$, $p = 0.017$; DoC: $Chi\text{-}square_{(2, 87)} = 12.76$, $p = 0.001$). In the sleep experiment, the classification performance of model 1 (source domain: sleep) and model 2 (source domain: sleep + anesthesia) was higher than that of model 3 (source domain: sleep + DoC) in sleep. Under anesthesia, there was a higher performance in model 1 (source domain: anesthesia) and model 2 when compared to model 4 (source domain: anesthesia + DoC). Finally, while considering the patients with DoC, performance in model 3 and model 4 was higher than that in model 5 (source domain: sleep + anesthesia + DoC). However, no significant differences in classification accuracy were observed for the state of awareness (sleep: $Chi\text{-}square_{(3, 20)} = 1.21$, $p = 0.751$; anesthesia: $Chi\text{-}square_{(3, 60)} = 5.49$, $p = 0.139$; DoC: $Chi\text{-}square_{(3, 116)} = 3.06$, $p = 0.382$) (Supplementary Table S4).

In resting-state EEG data (without TMS), we also classified low or high in both arousal and awareness using domain transfer learning (Table 3). For arousal, the classification performance using only anesthesia was higher when compared to anesthesia with DoC as a source domain in the training phase ($t_{(14)} = 7.242$, $p < 0.001$). However, for awareness, there were no significant differences in

classification accuracy when only the anesthesia and the combined anesthesia with DoC domains were used as the source domain ($t_{(14)} = 0.708$, $p = 0.490$). In patients with DoC, only high arousal existed; therefore, classification performance was overfitted when trained on the DoC domain only. In addition, there were no significant differences in classification accuracy between using only DoC and DoC with the anesthesia domain as source domains for awareness ($t_{(29)} = –0.322$, $p = 0.749$). This was the same result as the classification performance of TMS–EEG data using domain transfer learning, given the absence of sleep data.

Supplementary Note 5: Range of ECI and PCI in arousal and awareness

The optimal cutoff for ECI^{aro} and ECI^{awa} was 0.5 in all conditions. During sleep, ECI^{aro} ranged from 0.001 to 0.145 in NREM sleep, from 0.072 to 0.460 in REM sleep, and from 0.587 to 0.982 in healthy wakefulness. In addition, ECI^{awa} ranged from 0.001 to 0.462 in NREM sleep, between 0.519 and 0.989 in REM sleep, and between 0.893 and 0.996 in healthy wakefulness. Under anesthesia, the range of ECI^{aro} was between 0.571 and 0.945 for high state (wakefulness) and between 0.036 and 0.394 for low state (ketamine-, propofol-, and xenon-induced anesthesia). Similarly, the range of ECI^{awa} was from 0.568 to 0.976 for high state (wakefulness and ketamine-induced anesthesia) and from 0.030 to 0.466 for low state (propofol- and xenon-induced anesthesia). Finally, ECI^{aro} and ECI^{awa} in MCS patients ranged between 0.573 and 0.995 and 0.551 and 0.930, respectively. In addition, in UWS patients, ECI^{aro} ranged from 0.527 to 0.968 and ECI^{awa} ranged from 0.062 to 0.473. Consequently, low and high states were perfectly distinguished by an optimal cutoff of 0.5 in both ECI^{aro} and ECI^{awa}.

In ECI using resting-state EEG data, the optimal cutoff was 0.5 as with ECI using TMS–EEG data. Under anesthesia, the range of ECI^{aro} was between 0.771 and 0.993 for the high state (wakefulness) and between 0.001 and 0.456 for the low state (ketamine-, propofol-, and xenon-induced anesthesia). Similarly, ECI^{awa} ranged from 0.563 to 0.999 in the high state (wakefulness and

ketamine-induced anesthesia) and from 0.015 to 0.421 in the low state (propofol- and xenon-induced anesthesia). In patients with DoC, ECI^{aro} ranged between 0.589 and 0.995 for MCS and UWS patients. Finally, ECI^{awa} ranged between 0.520 and 0.985 in MCS patients and between 0.045 and 0.468 in UWS patients.

We calculated PCI in TMS–EEG sessions. During sleep, PCI varied from 0.100 to 0.293 in NREM sleep, from 0.315 to 0.522 in REM sleep with subjective experience, and from 0.440 to 0.668 in healthy wakefulness. PCI ranged from 0.125 to 0.273 under propofol- and xenon-induced anesthesia and from 0.388 to 0.678 in ketamine-induced anesthesia and wakefulness. Finally, in the severely brain-injured patients, PCI ranged between 0.120 and 0.300 for the patients with UWS and between 0.342 and 0.620 for MCS patients. In the four MCS* patients, the range of PCI was between 0.416 and 0.505.

Supplementary Note 6: Performance on ECI with only a few trials

Figure 5 shows the classification performance from a single trial up to the standard number of trials in anesthesia and patients with DoC. In anesthesia and wake conditions, calculating ECI with single trials achieved performance of 0.742 specificity, 0.917 sensitivity, and 0.885 AUC. Sensitivity was always above 0.9 even with a single trial, and AUC was above 0.9 from 2 trials. The specificity, on the other hand, was above 0.9 from 9 trials. However, as the number of trials increases, the performance did not improve (not higher than 0.9), and it sometimes fell below 0.9. However, it was always higher than 0.821. In patients with DoC, a specificity of 0.717, sensitivity of 0.806, and AUC of 0.740 were obtained using single trials. As a result, specificity, sensitivity, and AUC were above 0.9 from 13 trials, 7 trials, and 4 trials, respectively.

Supplementary Methods

Classification performance according to the number of trials in the ECI calculation

Originally, ECI is calculated by averaging the interclass probability of all trials in a single session. Here, the ECI was computed when changing the number of trials in a single session, from 1 to 80 trials, to explore the performance of the ECI according to the number of trials. For example, if a single session consisted of five trials, the average value of five trials was calculated as ECI.

Difference between correct and incorrect trials

We compared difference between correct and incorrect trials for all participants during sleep and healthy wakefulness in terms of spatiotemporal information. TMS-evoked amplitude from 200 to 400 ms was averaged over frontal, temporal, and parietal regions and compared using a paired t-test with Bonferroni correction. The division of each region was the same as when calculating relevance scores for a specific region.

Classification performance using EEG signals excluding frontal or parietal electrodes

We additionally calculated ECI using EEG signals excluding frontal or parietal electrodes. When converting to 2D meshes, in the case of excluding frontal or parietal electrodes, zero was added to the corresponding positions: frontal (Fp1-2, Fpz, AF1-2, AFz, F1-8, and Fz) and parietal (CP1-6, CPz, P1-4, P7-8, and Pz) regions. Then, two-class classification was performed based on CNN-DR2 as before. Then, ECI was calculated in each single session.

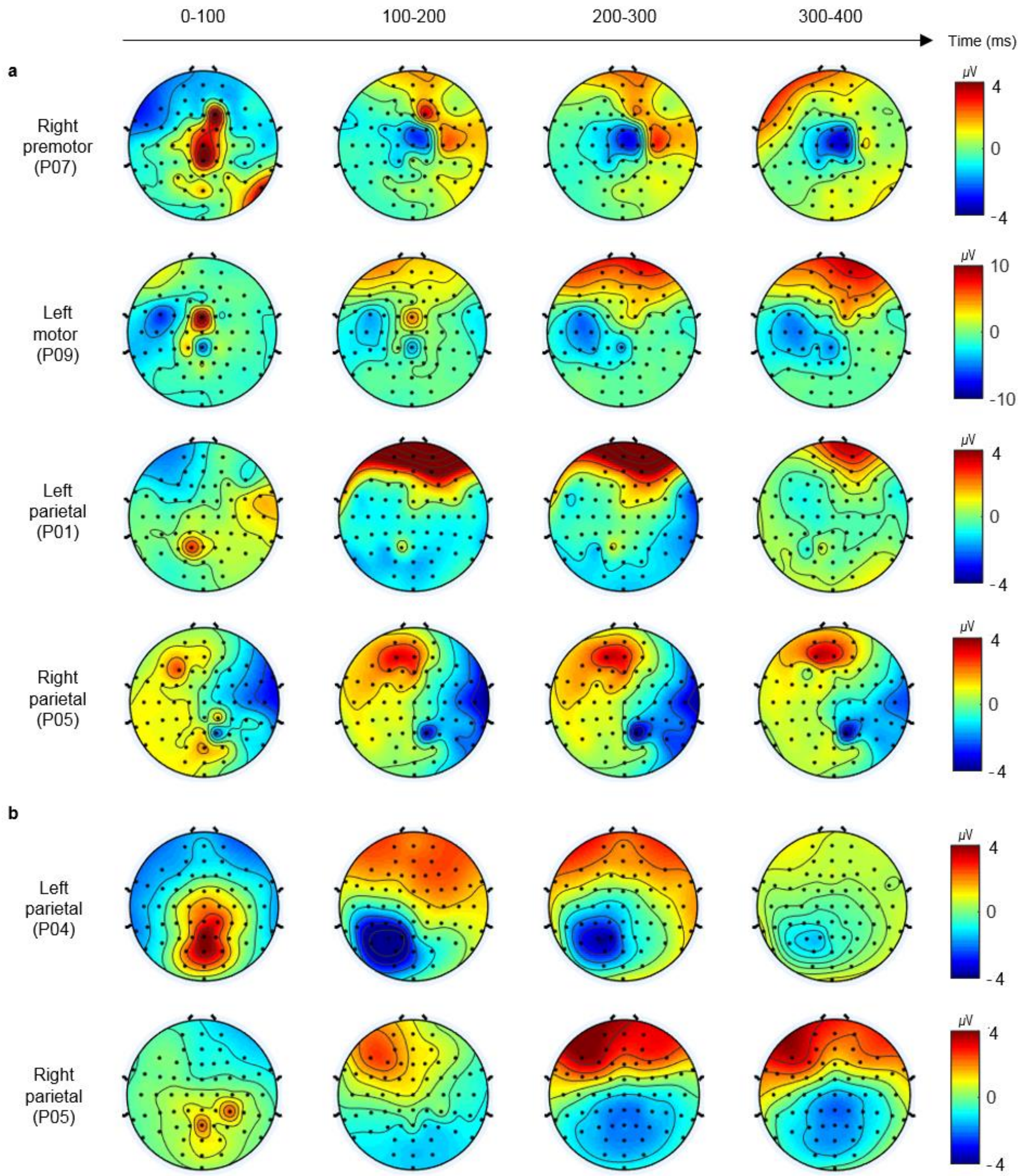

Supplementary Figure 1. TMS-evoked responses with different TMS target sites: TMS-evoked changes are depicted by intervals of 100 ms in (a) UWS patients using four different TMS target sites (right premotor, left motor, left parietal, and right parietal regions) and (b) healthy participants during NREM sleep with no dreams using two different TMS target sites (left and right parietal regions).

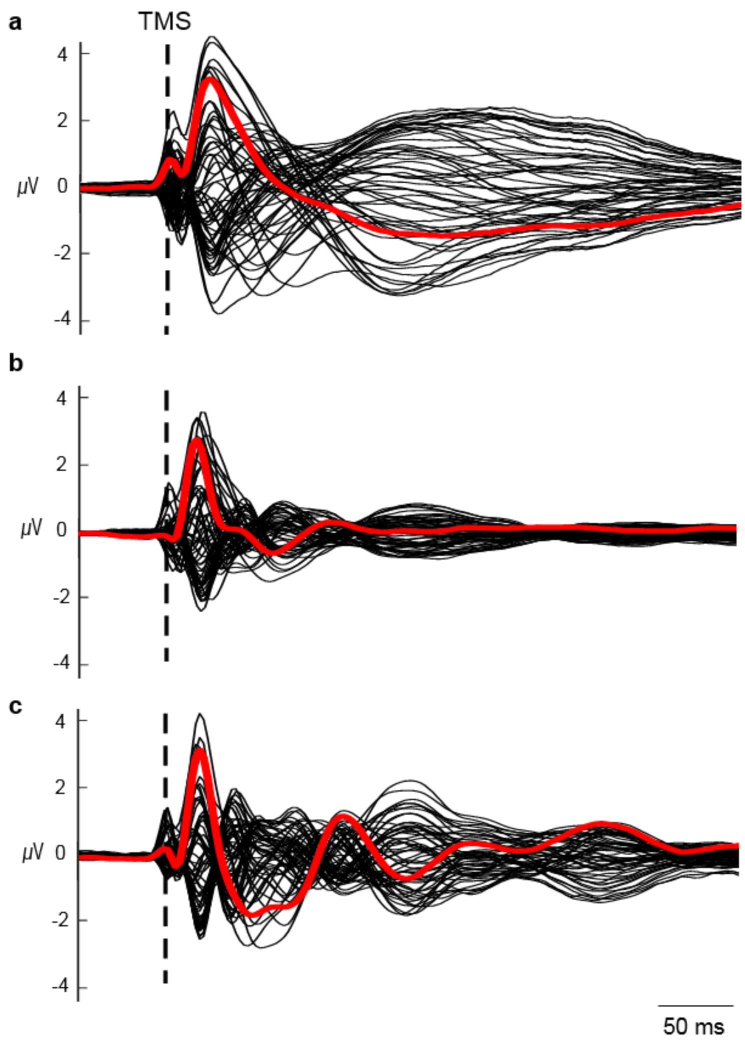

Supplementary Figure 2. Averaged TMS-evoked responses for the 60 channels in all participants in sleep and wakefulness: (a) NREM sleep with no subjective experience, (b) REM sleep with subjective experience, and (c) healthy wakefulness. Of the six participants, P01 and P04 were stimulated on the left superior parietal cortex and their data were flipped. The black traces indicate the voltage measured from 59 electrodes and the red trace indicates the voltage for the electrode under the coil (superior parietal cortex) in −100–400 ms. The vertical dotted line indicates the time of the TMS.

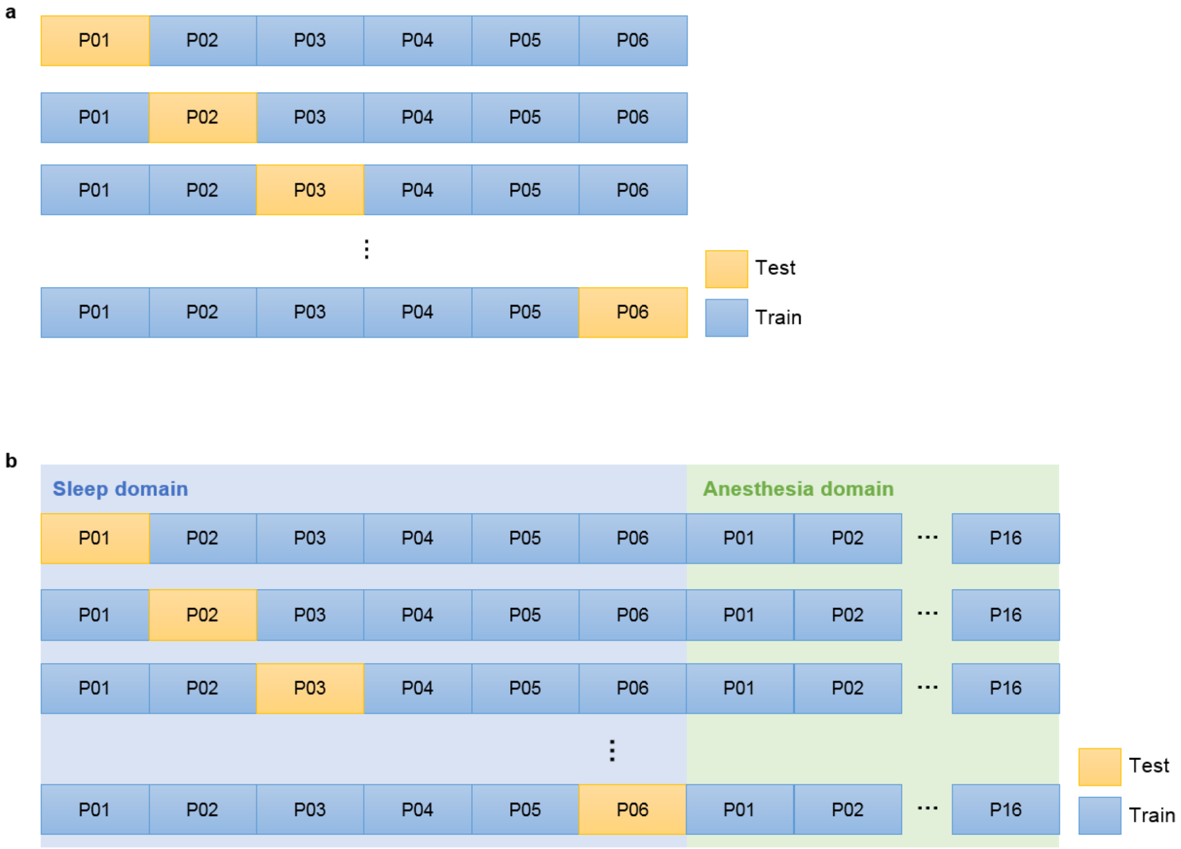

Supplementary Figure 3. Application of leave-one-participant-out method: (a) In a single domain, data of one participant are used as test data, and the remaining data are used as training data. For example, in the sleep domain with a total of six participants, if data of one participant are used as test data, the data of the remaining five participants are used as training data. (b) In domain transfer learning, if the source domains are sleep and anesthesia and the target domain is sleep, one participant in the sleep domain is used as test data, while all participants in the anesthesia and sleep domains were used for training, excluding the one participant used as test data.

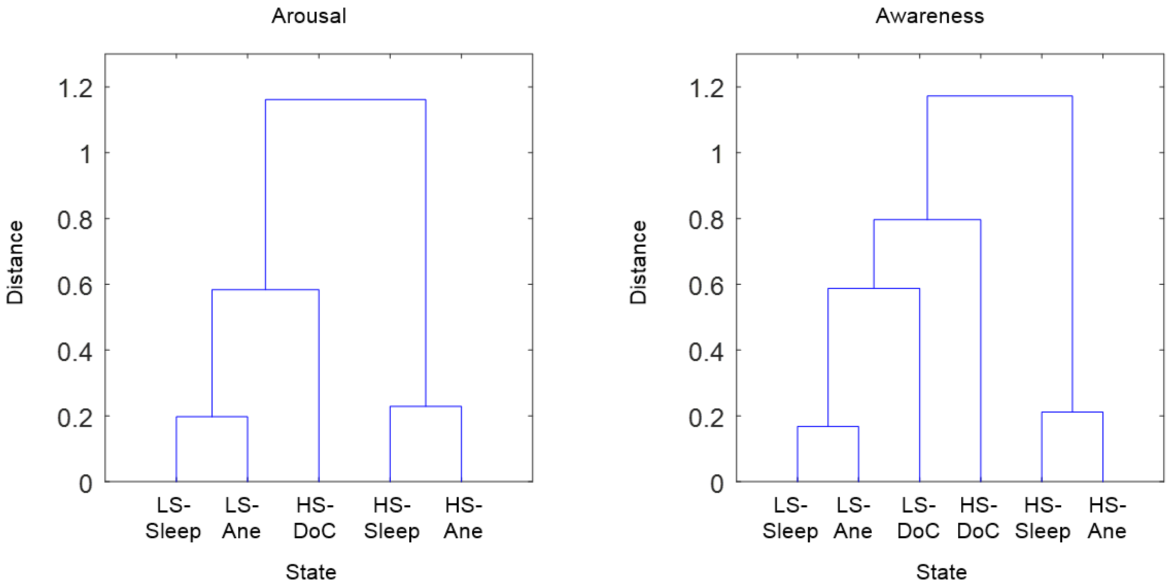

Supplementary Figure 4. Hierarchical clustering on TMS-induced responses in physiological, pharmacological, and pathological conditions: We calculated the cosine distance between the averaged TMS-evoked potentials (the 200–400 ms period after TMS) of low and high states of arousal (left) and awareness (right) in the three conditions. In hierarchical clustering, the *y*-axis indicates the cosine distance. As patients with DoC have high arousal, there is no LS-DoC group in arousal.

LS-Sleep = low state in sleep; LS-Ane = low state in anesthesia; LS-DoC = low state in DoC; HS-Sleep = high state in sleep; HS-Ane = high state in anesthesia; HS-DoC = high state in DoC.

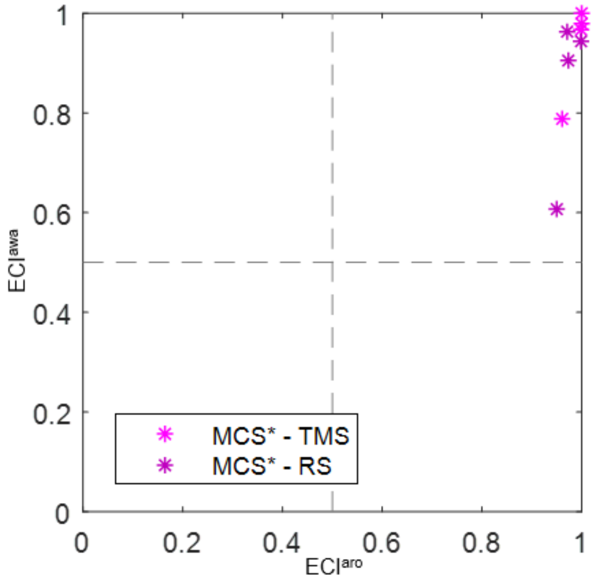

Supplementary Figure 5. ECI for MCS* patients: Each sign in ECI represents the average value of each session and the gray dotted lines represent ECI cutoff. In TMS–EEG data (TMS) and resting-state EEG data (RS), four MCS* patients are represented with cross marks and asterisks, respectively.

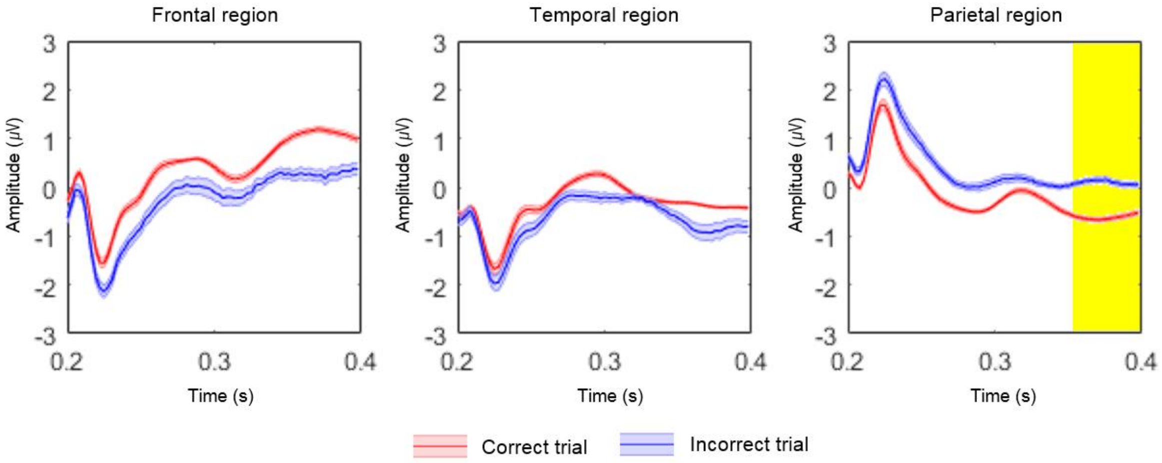

Supplementary Figure 6. Difference between correct and incorrect trials for awareness during sleep and healthy wakefulness: Temporal difference between correct (red line) and incorrect (blue line) trials was investigated over frontal, temporal, and parietal regions. The shaded line indicates the amplitude from 0.2 to 0.4 s after TMS. The measure of centre means an averaged amplitude and error bars indicate standard error in all participants. Shaded yellow box indicates the significant difference with Bonferroni correction.

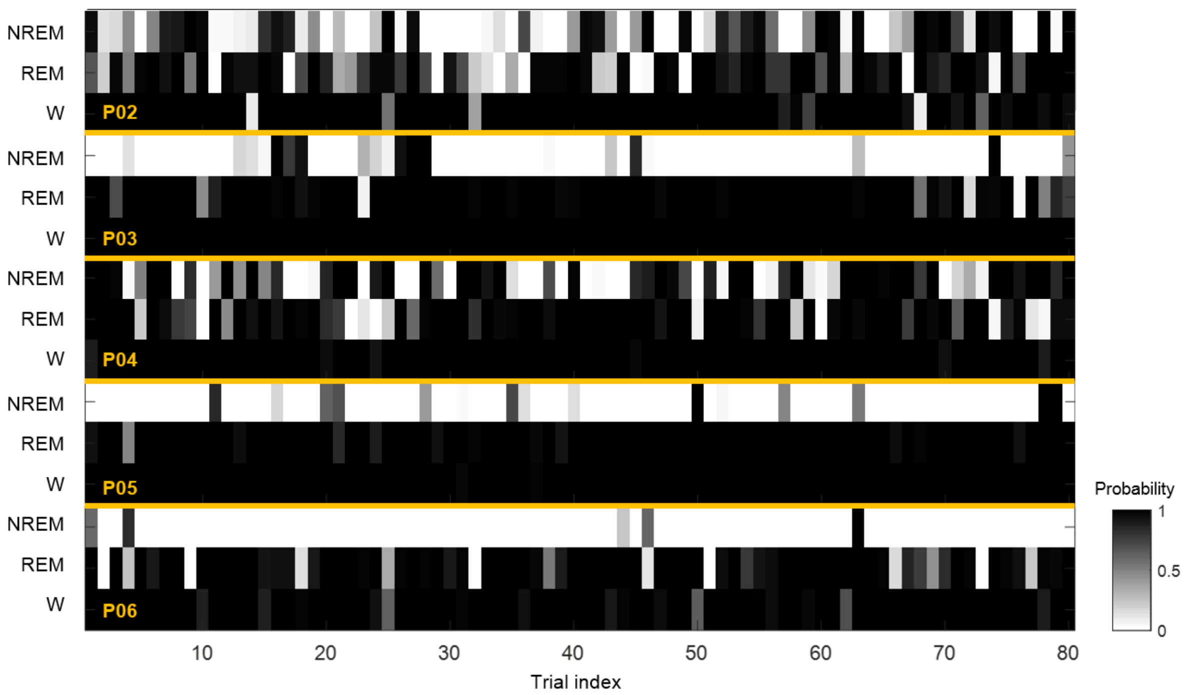

Supplementary Figure 7. Interclass probability in single trials for ECI^{awa} under sleep and healthy wakefulness: Each colored box indicates the probability that the corresponding trial is high in each participant. If it was a perfect prediction in one trial, during sleep and healthy wakefulness, NREM sleep with no subjective experience (low awareness) has a probability of less than 0.5, whereas REM sleep and normal wakefulness (high awareness) have probabilities of more than 0.5.

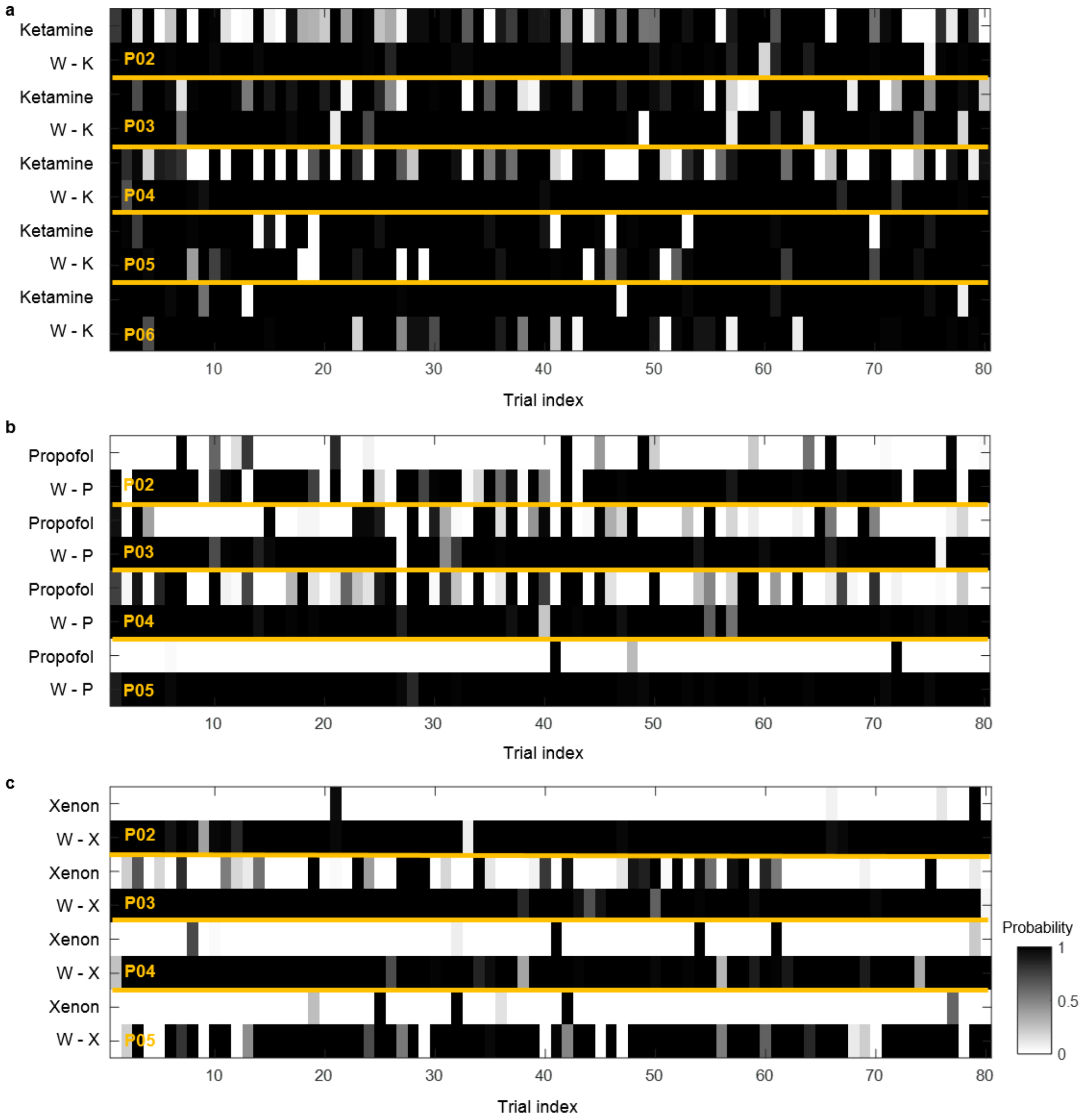

Supplementary Figure 8. Interclass probability in single trials for ECI^{awa} under anesthesia and wakefulness before anesthesia at the participant level: Anesthesia is (a) ketamine (P02 to P06), (b) propofol (P02 to P05), and (c) xenon (P02 to P05). Each colored box indicates the probability that the corresponding trial is high in each participant. Wakefulness before anesthesia and anesthesia with ketamine are classified as high awareness, whereas anesthesia with propofol and xenon are classified as low awareness.

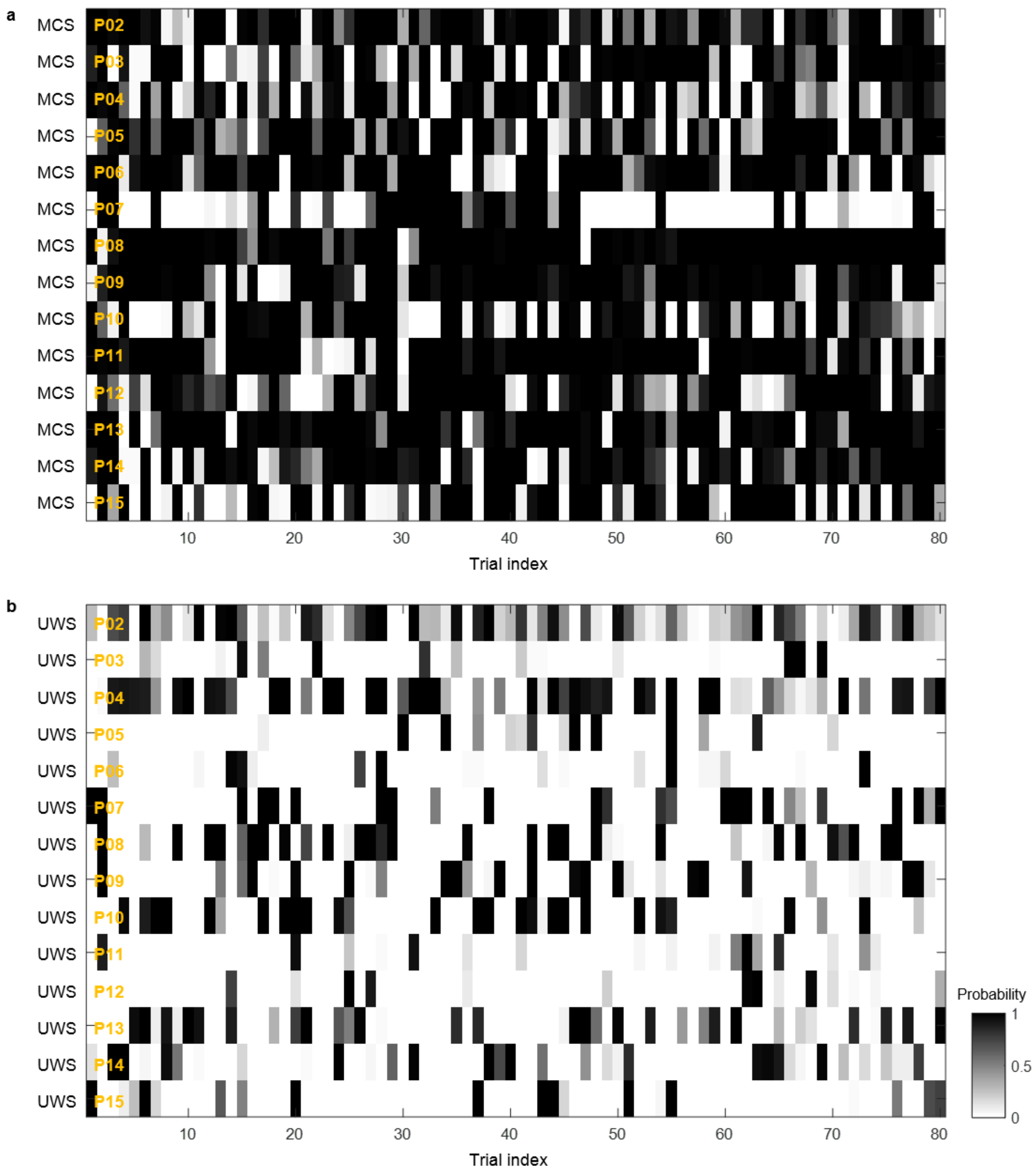

Supplementary Figure 9. Interclass probability in single trials for ECI^{awa} in patients with DoC: Each colored box indicates the probability that the corresponding trial is high in each participant. (a) MCS (P02 to P15) and (b) UWS patients (P02 to P15) are classified as high and low awareness, respectively.

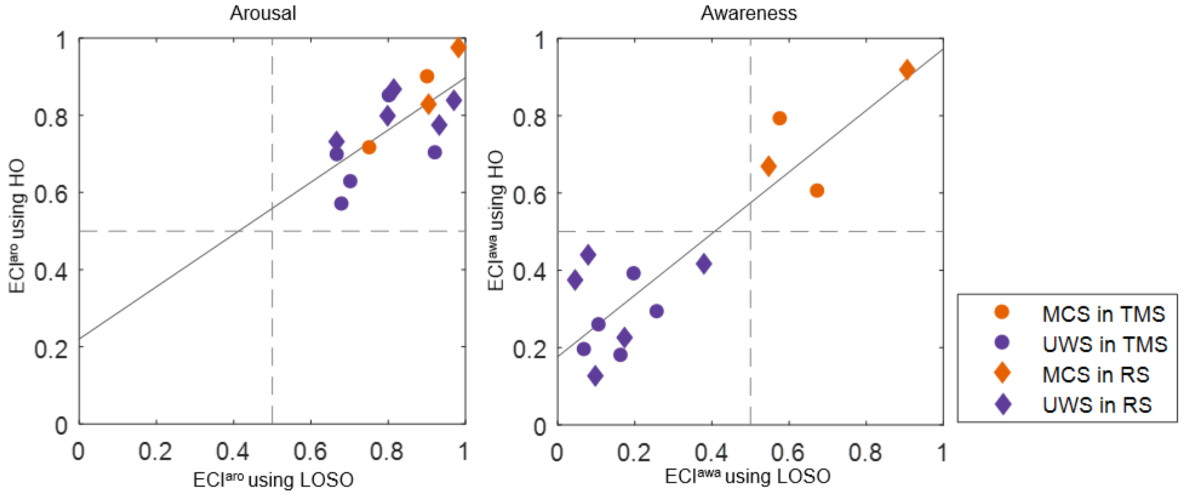

Supplementary Figure 10. Relationship between ECI using leave-one-participant-out and hold-out approach. The *x* axis represents ECI using leave-one-participant-out (LOPO), and the *y*-axis represents ECI using hold-out (HO) approach for arousal (left) and awareness (right). The gray dashed lines indicate the optimal cutoff (0.5) dividing the space into high and low states of ECI. Orange and purple respectively indicate MCS and UWS patients, while dots and diamonds refer to TMS–EEG (TMS) and resting-state (RS) EEG data. The solid lines represent linear fits to the data.

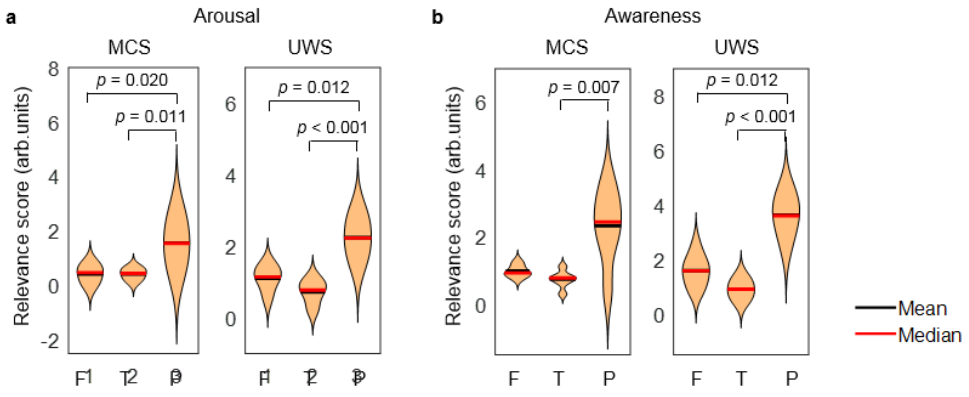

Supplementary Figure 11. Relevance scores from LRP in TMS–EEG data with only non-parietal stimulation in the patients with DoC: (a) arousal and (b) awareness. Six out of 15 MCS patients and eight out of 15 UWS patients applied TMS stimulation in other regions than the parietal cortex. The violin plots depict averaged relevance scores over the frontal, temporal, and parietal regions in each participant. The exact *p*-value corresponding to the significance level was shown with Fisher's least significant differences method for multiple comparisons. [arb. units] denotes an arbitrary unit.

F = frontal region; T = temporal region; P = parietal region.

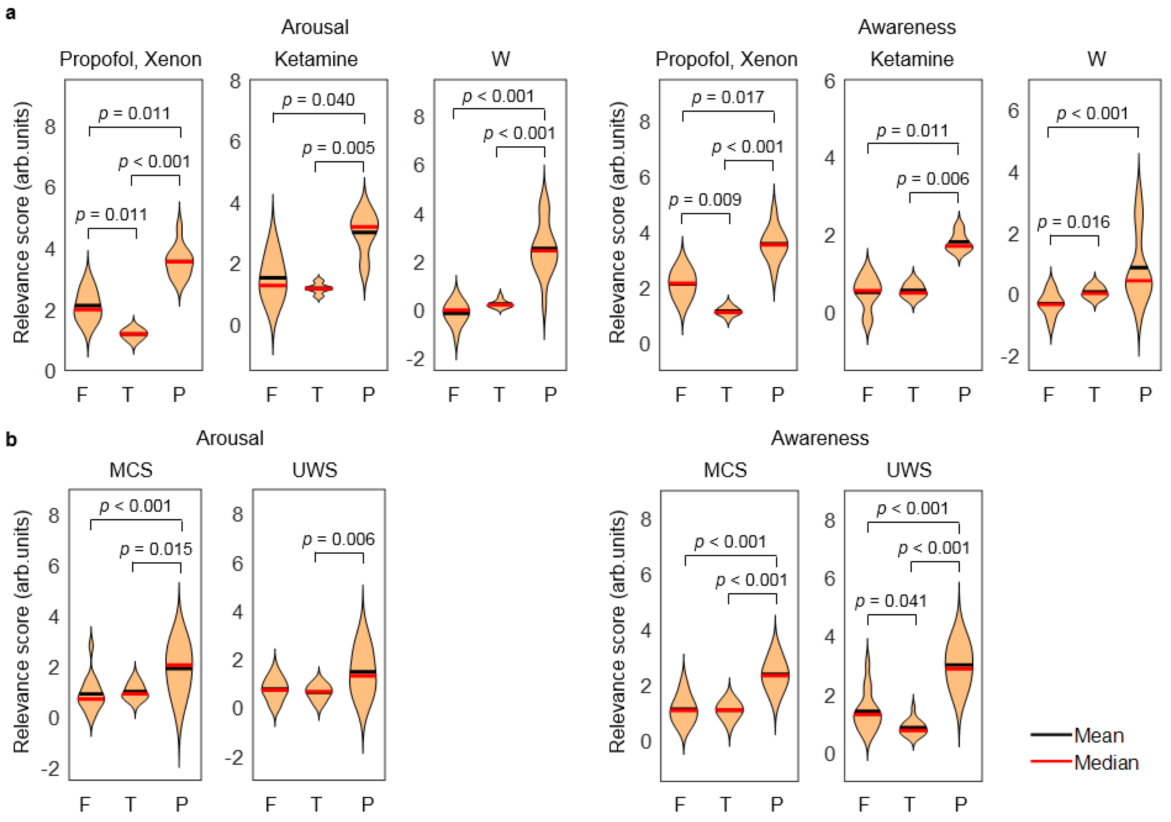

Supplementary Figure 12. Relevance scores from LRP in resting-state EEG data: (a) anesthesia /wake and (b) patients with DoC. The violin plots depict averaged relevance scores over the frontal, temporal, and parietal regions in each participant. The exact *p*-value corresponding to the significance level was shown with Fisher's least significant differences method for multiple comparisons. [arb. units] denotes an arbitrary unit.

F = frontal region; T = temporal region; P = parietal region.

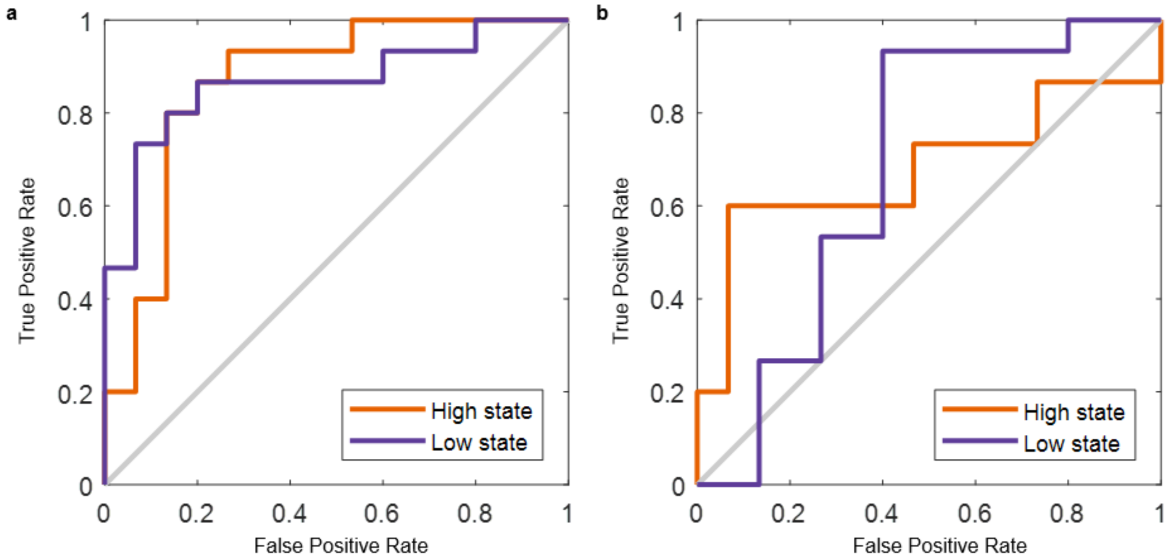

Supplementary Figure 13. Receiver operating characteristic curve analysis for ECI^{awa} in patients with DoC using TMS–EEG data: Classification performance was calculated by (a) excluding frontal electrodes and (b) excluding parietal electrodes.

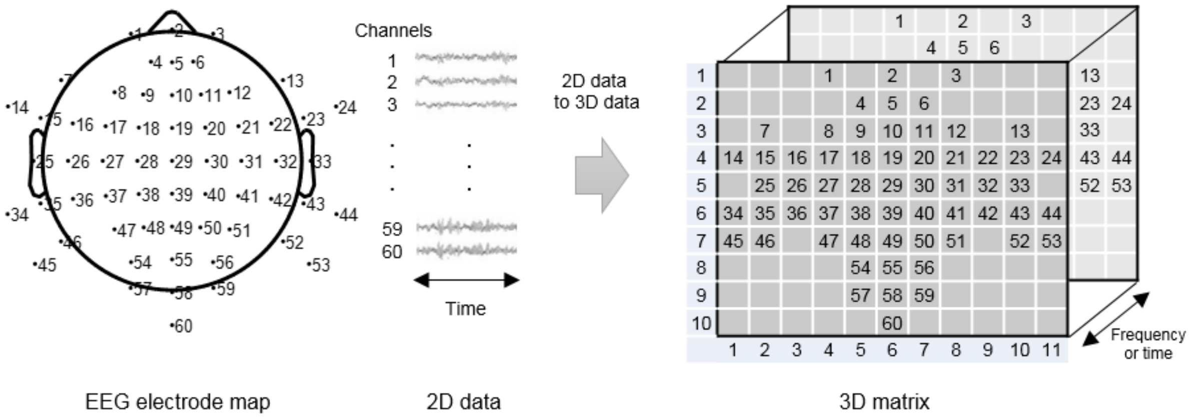

Supplementary Figure 14. Data input for ECI framework: The time-series 2D EEG data were converted to 3D features utilizing the EEG electrode map. According to spatial information, the 10 × 11 matrix was transformed. Zero was entered for the *null* electrode position. The *z* axis represents frequency or time information. As for spectral information, the frequency indicated the delta, theta, alpha, beta, and gamma bands. As for temporal information, the time period of 200–400 ms after the TMS stimulation was considered. Thus, the input was a 10 × 11 × 5 matrix (DR1; spatio-spectral information) or 10 × 11 × 72 matrix (DR2; spatiotemporal information).

Supplementary Table 1. Classification accuracy (%) during sleep and healthy wakefulness: For both arousal and awareness, two-class (low versus high) classification was performed using three classifiers and two data inputs. The two-sided multiple *t*-tests were used for post-hoc analysis using Fisher's least significant differences method for multiple comparisons.

	Arousal						Awareness					
Classifier	LDA		SVM		CNN		LDA		SVM		CNN	
Input	DR1	DR2	DR1	DR2	DR1	DR2	DR1	DR2	DR1	DR2	DR1	DR2
P01	77.23	60.14	49.49	57.17	49.49	87.98	86.62	60.37	68.59	72.83	31.82	96.26
P02	61.28	59.25	76.62	77.26	76.62	86.88	63.80	61.22	58.98	79.62	74.07	86.55
P03	50.50	46.06	72.96	74.26	72.96	91.49	60.06	59.68	41.82	71.58	73.24	92.71
P04	71.21	59.78	82.70	80.78	82.70	89.02	66.80	56.37	44.26	78.78	53.04	84.51
P05	61.39	54.70	58.23	73.34	58.28	88.20	58.62	58.58	65.90	75.96	74.96	96.47
P06	65.86	66.07	67.97	79.66	67.97	83.18	68.50	60.15	70.37	79.71	88.04	95.19
Mean ±SD	64.58 ±9.22	57.67 ±6.74	67.99 ±12.27	73.75 ±8.62	68.00 ±12.26	**87.79 ±2.74**	66.91 ±9.02	59.40 ±1.72	58.32 ±12.48	76.43 ±3.57	65.86 ±20.10	**91.95 ±5.19**
p-value	*0.002*	*<0.001*	*0.005*	0.051	*0.005*	-	*0.004*	*<0.001*	*<0.001*	0.154	*0.011*	-

LDA = linear discriminant analysis; SVM = support vector machine; CNN = convolutional neural network; DR1 = 10 × 11 × 5 (spatio-spectral information); DR2 = 10 × 11 × 72 (spatiotemporal information); *p*-value = statistics with CNN-DR2 using Fisher's least significant differences method for multiple comparisons.

Supplementary Table 2. Statistics of classification performance under sleep data for post-hoc analysis: The *p*-value was determined using two-sided multiple *t*-tests with Fisher's least significant differences method for multiple comparisons, and a *p*-value less than 0.05 is indicated in italics.

	Arousal			Awareness		
Classifier-Input	95% CI		*p*-value	95% CI		*p*-value
	Lower limit	Upper limit		Lower limit	Upper limit	
LDA-DR1 vs. LDA-DR2	-6.085	17.752	0.337	-5.755	18.089	0.311
LDA-DR1 vs. SVM-DR1	-14.002	9.835	0.732	-7.255	16.589	0.443
LDA-DR1 vs. SVM-DR2	-19.252	4.585	0.228	-20.922	2.922	0.139
LDA-DR1 vs. CNN-DR1	-14.168	9.668	0.711	-14.089	9.755	0.722
LDA-DR1 vs. CNN-DR2	-31.085	-7.249	*0.002*	-29.589	-5.745	*0.004*
LDA-DR2 vs. SVM-DR1	-19.835	4.002	0.193	-13.422	10.422	0.805
LDA-DR2 vs. SVM-DR2	-25.085	-1.249	*0.030*	-27.089	-3.245	*0.013*
LDA-DR2 vs. CNN-DR1	-20.002	3.835	0.184	-20.255	3.589	0.171
LDA-DR2 vs. CNN-DR2	-36.918	-13.082	*<0.001*	-35.755	-11.911	*<0.001*
SVM-DR1 vs. SVM-DR2	-17.168	6.668	0.388	-25.589	-1.745	*0.025*
SVM-DR1 vs. CNN-DR1	-12.085	11.752	0.978	-18.755	5.089	0.261
SVM-DR1 vs. CNN-DR2	-29.002	-5.165	*0.005*	-34.255	-10.411	*<0.001*
SVM-DR2 vs. CNN-DR1	-6.835	17.002	0.403	-5.089	18.755	0.261
SVM-DR2 vs. CNN-DR2	-23.752	0.085	0.051	-20.589	3.255	0.154
CNN-DR1 vs. CNN-DR2	-28.835	-4.999	*0.005*	-27.422	-3.578	*0.011*

95% CI = 95% confidence interval for the difference in the group means.

Supplementary Table 3. Statistics of classification performance for the data of patients with disorders of consciousness with post-hoc analysis: CNN-DR2 was used as the classifier and input. In DR2, the time periods of the temporal information were generated at 200 ms intervals after the TMS. The *p*-value was measured using two-sided multiple *t*-tests with Fisher's least significant differences method for multiple comparisons, and a *p*-value less than 0.05 is indicated in italics.

	Awareness		
Temporal information	95% CI		*p*-value
	Lower limit	Upper limit	
0–200 ms vs. 200–400 ms	-35.787	-9.346	*<0.001*
0–200 ms vs. 400–600 ms	-13.554	12.887	0.960
200–400 ms vs. 400–600 ms	9.012	35.454	*<0.001*

Supplementary Table 4. Statistics of classification performance in transfer learning for post-hoc analysis: The *p*-value was performed using two-sided multiple *t*-tests with Fisher's least significant differences method for multiple comparisons, and a *p*-value less than 0.05 is indicated in italics. In the patient, performance using model 1 was overfitted; therefore, it is not included in the comparison.

		Arousal			Awareness		
Domain	Model comparison	95% CI Lower limit	95% CI Upper limit	*p*-value	95% CI Lower limit	95% CI Upper limit	*p*-value
Sleep	Model 1 vs. model 2	-6.668	9.334	0.744	-4.501	11.501	0.391
	Model 1 vs. model 3	2.165	18.168	*0.012*	-3.834	12.168	0.307
	Model 1 vs. model 5	-0.834	15.168	0.079	-5.668	10.334	0.567
	Model 2 vs. model 3	0.831	16.834	*0.030*	-7.334	8.668	0.870
	Model 2 vs. model 5	-2.168	13.834	0.153	-9.168	6.834	0.775
	Model 3 vs. model 5	-11.001	5.001	0.462	-9.834	6.168	0.653
Anesthesia	Model 1 vs. model 2	-16.558	9.245	0.578	-26.526	-0.723	*0.038*
	Model 1 vs. model 4	2.879	28.683	*0.016*	-18.464	7.339	0.398
	Model 1 vs. model 5	-5.901	19.901	0.287	-25.089	0.714	0.064
	Model 2 vs. model 4	6.535	32.339	*0.003*	-4.839	20.964	0.220
	Model 2 vs. model 5	-2.245	23.558	0.105	-11.464	14.339	0.827
	Model 4 vs. model 5	-21.683	4.120	0.182	-19.526	6.276	0.314
Patients with DoC	Model 1 vs. model 3	-	-	-	-27.970	7.236	0.248
	Model 1 vs. model 4	-	-	-	-13.703	21.503	0.664
	Model 1 vs. model 5	-	-	-	-24.203	11.003	0.462
	Model 3 vs. model 4	-2.887	23.554	0.125	-3.336	31.870	0.112
	Model 3 vs. model 5	0.462	26.904	*0.042*	-13.836	21.370	0.674
	Model 4 vs. model 5	10.796	37.237	*<0.001*	-28.103	7.103	0.242

Model 1 = single domain (sleep or anesthesia or DoC); model 2 = double domain (sleep + anesthesia); model 3 = double domain (sleep + DoC); model 4 = double domain (anesthesia + DoC); model 5 = triple domain (sleep + anesthesia + DoC).

Supplementary Table 5. Statistics of relevance scores from LRP in TMS–EEG data: The relevance scores over frontal, temporal, and parietal regions were compared using the Kruskal–Wallis test. A *p*-value less than 0.05 is indicated in italics.

Condition	State	Arousal		Awareness	
		Chi-square	*p*-value	Chi-square	*p*-value
Sleep	NREM	14.75	*<0.001*	9.56	*0.008*
	REM	13.66	*0.001*	10.15	*0.006*
	W	11.47	*0.003*	13.66	*0.001*
Anesthesia	PPF, xenon	22.72	*<0.001*	25.06	*<0.001*
	Ketamine	11.94	*0.003*	11.42	*0.003*
	W before A	31.81	*<0.001*	30.64	*<0.001*
Patients with DoC	MCS	16.37	*<0.001*	16.09	*<0.001*
	UWS	29.01	*<0.001*	34.42	*<0.001*

NREM = non-rapid eye movement sleep with no subjective experience; REM = rapid eye movement sleep with subjective experience; W = healthy wakefulness, PPF = propofol; W before A = wakefulness before anesthesia; MCS = patients in minimally conscious state; UWS = patients with unresponsive wakefulness syndrome.

Supplementary Table 6. Statistics of relevance scores from LRP in TMS–EEG data with only non-parietal stimulation: Six out of 15 MCS patients and eight out of 15 UWS patients applied TMS stimulation in other regions than the parietal cortex. We compared the relevance scores over frontal, temporal, and parietal regions with only non-parietal stimulation using the Kruskal–Wallis test. A *p*-value less than 0.05 is indicated in italics.

Condition	State	Arousal		Awareness	
		Chi-square	*p*-value	Chi-square	*p*-value
Patients with DoC	MCS	7.94	*0.019*	7.31	*0.026*
	UWS	16.84	*<0.001*	17.65	*<0.001*

MCS = patients in minimally conscious state; UWS = patients with unresponsive wakefulness syndrome.

Supplementary Table 7. Statistics of relevance scores from LRP in resting-state EEG data: The relevance scores over the frontal, temporal, and parietal regions were compared using the Kruskal–Wallis test. A *p*-value less than 0.05 is indicated in italics.

Condition	State	Arousal		Awareness	
		Chi-square	*p*-value	Chi-square	*p*-value
Anesthesia	PPF, Xenon	25.81	*<0.001*	25.06	*<0.001*
	Ketamine	8.54	0.014	9.42	*0.009*
	W before A	31.03	*<0.001*	16.98	*<0.001*
Patients with DoC	MCS	11.83	*0.003*	26.55	*<0.001*
	UWS	8.03	*0.018*	29.98	*<0.001*

W before A = wakefulness before anesthesia.

Supplementary Table 8. Number of sessions and trials for all participants during sleep and healthy wakefulness to calculate ECI: The range indicates the number of trials in one session within each state.

Participant	TMS target site	State	Number of sessions	Number of trials (range)
P01	Left parietal	NREM	3	315 (37–199)
		REM	1	175
		W	3	500 (145–190)
P02	Right parietal	NREM	12	1919 (13–272)
		REM	6	1299 (103–257)
		W	4	982 (233–263)
P03	Right parietal	NREM	8	1488 (21–256)
		REM	5	363 (24–130)
		W	3	686 (182–258)
P04	Left parietal	NREM	12	2062 (15–273)
		REM	8	997 (12–255)
		W	3	640 (199–229)
P05	Right parietal	NREM	5	920 (91–249)
		REM	4	417 (13–138)
		W	4	959 (225–260)
P06	Right parietal	NREM	6	671 (42–163)
		REM	6	602 (21–117)
		W	3	600 (163–243)

Supplementary Table 9. Target TMS site and the number of trials in all participants under anesthesia to calculate ECI and PCI: The TMS target site is the same in wakefulness (W) before anesthesia and under anesthesia.

State	Participant	TMS target site	Number of trials	
			W before anesthesia	Anesthesia
Ketamine	P01	Left parietal	232	218
	P02	Left parietal	295	240
	P03	Left parietal	257	226
	P04	Left parietal	228	279
	P05	Left parietal	215	244
	P06	Left parietal	209	244
Propofol	P01	Left parietal	206	341
	P02	Left parietal	194	236
	P03	Left parietal	209	186
	P04	Left parietal	194	279
	P05	Left parietal	194	228
Xenon	P01	Left parietal	172	164
	P02	Left motor	202	206
	P03	Left parietal	285	204
	P04	Left motor	127	138
	P05	Left parietal	152	163

Supplementary Table 10. Demographical data in severely brain-injured patients.

State	Participant	Gender	Time since injury	Etiology
MCS	P01	M	9 months	stroke
	P02	M	1 month	stroke
	P03	F	1 month	stroke
	P04	M	1 month	stroke
	P05	M	52 months	TBI
	P06	F	50 months	TBI
	P07	F	36 months	TBI
	P08	M	6 months	anoxia
	P09	M	74 months	anoxia
	P10	M	169 months	anoxia
	P11	M	158 months	TBI
	P12	M	343 months	TBI + anoxia
	P13	M	14 months	TBI
	P14	M	56 months	TBI
	P15	F	2 months	anoxia
UWS	P01	F	8 months	TBI
	P02	F	1 month	anoxia
	P03	M	1 month	TBI
	P04	F	47 months	TBI
	P05	M	6 months	TBI
	P06	F	1 month	stroke
	P07	M	4 months	anoxia
	P08	F	1 month	stroke
	P09	F	13 months	TBI
	P10	M	13 months	TBI
	P11	M	13 months	TBI
	P12	M	3 months	TBI
	P13	F	2 weeks	stroke
	P14	M	6 months	TBI
	P15	M	1 month	stroke
MCS*	P01	M	11 months	TBI
	P02	F	3 months	stroke
	P03	M	52 months	TBI
	P04	F	8 months	TBI + stroke

M = male, F = female, TBI = traumatic brain injury.

Supplementary Table 11. Target TMS site and the number of trials in severely brain-injured patients to calculate ECI and PCI.

State	Participant	TMS target site	Number of trials
MCS	P01	Left parietal	408
	P02	Left parietal	153
	P03	Left parietal	174
	P04	Left parietal	180
	P05	Left parietal	275
	P06	Right premotor	400
	P07	Left premotor	352
	P08	Right premotor	316
	P09	Left premotor	332
	P10	Left parietal	218
	P11	Right parietal	355
	P12	Right premotor	205
	P13	Right parietal	259
	P14	Right parietal	466
	P15	Left motor	243
UWS	P01	Left parietal	315
	P02	Right parietal	169
	P03	Right parietal	206
	P04	Right premotor	321
	P05	Right parietal	313
	P06	Left motor	187
	P07	Left premotor	452
	P08	Left parietal	215
	P09	Left premotor	301
	P10	Left premotor	311
	P11	Left parietal	133
	P12	Left parietal	158
	P13	Right motor	182
	P14	Right motor	156
	P15	Right motor	200
MCS*	P01	Left premotor	395
	P02	Left premotor	393
	P03	Right premotor	328
	P04	Left premotor	400

Supplementary Table 12. Number of trials in resting-state EEG under anesthesia to calculate ECI: W before anesthesia refers to healthy wakefulness before anesthetic injection.

State	Participant	Number of trials	
		W before anesthesia	Anesthesia
Ketamine	P01	297	307
	P02	308	299
	P03	623	255
	P04	433	325
	P05	117	266
Propofol	P01	272	86
	P02	1012	984
	P03	322	211
	P04	348	141
	P05	119	1062
Xenon	P01	307	268
	P02	361	347
	P03	299	316
	P04	302	283
	P05	295	299

Supplementary Table 13. Number of trials in resting-state EEG in severely brain-injured patients to calculate ECI.

State	Participant	Number of trials
MCS	P01	304
	P02	316
	P03	557
	P04	300
	P05	467
	P06	342
	P07	292
	P08	375
	P09	985
	P10	266
	P11	414
	P12	485
	P13	272
	P14	299
	P15	907
UWS	P01	332
	P02	281
	P03	347
	P04	290
	P05	515
	P06	283
	P07	385
	P08	348
	P09	323
	P10	248
	P11	288
	P12	853
	P13	250
	P14	845
	P15	849
MCS*	P01	427
	P02	318
	P03	307
	P04	304

Supplementary Table 14. CNN architecture.

Layer	Layer type	# Units	Unit type	Size	Stride	Output size
Input						(10, 11, x)
C1	Convolutional	100	ReLU	(3, 3)	(1, 1)	(8, 9, 100)
C2	Convolutional	80	ReLU	(2, 2)	(1, 1)	(7, 8, 80)
P1	Max-pooling			(2, 2)	(1, 2)	(6, 4, 80)
C3	Convolutional	60	ReLU	(3, 3)	(1, 1)	(4, 2, 60)
C4	Convolutional	40	ReLU	(2, 2)	(2, 1)	(2, 1, 40)
P2	Max-pooling			(2, 1)	(2, 1)	(1, 1, 40))
C5	Convolutional	2	ReLU	(1, 1)	(1, 1)	(1, 1, 2)
F1	Fully connected					2
S1	Softmax					2

ReLU = rectified linear unit; x = 10 × 11 × 5 (spatio-spectral information) or 10 × 11 × 72 (spatiotemporal information).
