# Supplementary material for: Quantifying arousal and awareness in altered states of consciousness using interpretable deep learning
Source: Nat Commun. 2022 Feb 25;13:1064. doi: 10.1038/s41467-022-28451-0 (PMC8881479; doi:10.1038/s41467-022-28451-0)
Supplement: Supplementary file 2 — Reporting summary [file 41467_2022_28451_MOESM2_ESM.pdf]

nature research

Corresponding author(s): Olivia Gosseries and Seong-Whan Lee

Last updated by author(s): Dec 6, 2021

Reporting Summary

Nature Research wishes to improve the reproducibility of the work that we publish. This form provides structure for consistency and transparency in reporting. For further information on Nature Research policies, see our Editorial Policies and the Editorial Policy Checklist.

Statistics

For all statistical analyses, confirm that the following items are present in the figure legend, table legend, main text, or Methods section.

n/a	Confirmed	
☐	☒	The exact sample size (n) for each experimental group/condition, given as a discrete number and unit of measurement
☐	☒	A statement on whether measurements were taken from distinct samples or whether the same sample was measured repeatedly
☐	☒	The statistical test(s) used AND whether they are one- or two-sided<br>*Only common tests should be described solely by name; describe more complex techniques in the Methods section.*
☐	☒	A description of all covariates tested
☐	☒	A description of any assumptions or corrections, such as tests of normality and adjustment for multiple comparisons
☐	☒	A full description of the statistical parameters including central tendency (e.g. means) or other basic estimates (e.g. regression coefficient) AND variation (e.g. standard deviation) or associated estimates of uncertainty (e.g. confidence intervals)
☐	☒	For null hypothesis testing, the test statistic (e.g. F, t, r) with confidence intervals, effect sizes, degrees of freedom and P value noted<br>*Give P values as exact values whenever suitable.*
☒	☐	For Bayesian analysis, information on the choice of priors and Markov chain Monte Carlo settings
☒	☐	For hierarchical and complex designs, identification of the appropriate level for tests and full reporting of outcomes
☐	☒	Estimates of effect sizes (e.g. Cohen's d, Pearson's r), indicating how they were calculated

Our web collection on statistics for biologists contains articles on many of the points above.

Software and code

Policy information about availability of computer code

Data collection	Nexstim eXimia (Nexstim Plc.) recording software
Data analysis	Matlab 2017a (MathWorks Inc.), using the SiSyPhus Project Matlab program (University of Milan, Italy) and custom code as well as function from the EEGLAB toolbox, the BBCI toolbox, and LRP toolbox

For manuscripts utilizing custom algorithms or software that are central to the research but not yet described in published literature, software must be made available to editors and reviewers. We strongly encourage code deposition in a community repository (e.g. GitHub). See the Nature Research guidelines for submitting code & software for further information.

Data

Policy information about availability of data

All manuscripts must include a data availability statement. This statement should provide the following information, where applicable:

- Accession codes, unique identifiers, or web links for publicly available datasets
- A list of figures that have associated raw data
- A description of any restrictions on data availability

All data analyzed during this study are available on reasonable request to the corresponding author and resting-state EEG signals during anesthesia and wake are made available online upon request at the Zenodo repository (https:// doi.org/10.5281/zenodo.806176). Part of the data will also be freely available through EBRAIN.

Field-specific reporting

Please select the one below that is the best fit for your research. If you are not sure, read the appropriate sections before making your selection.

- [x] Life sciences
- [ ] Behavioural & social sciences
- [ ] Ecological, evolutionary & environmental sciences

For a reference copy of the document with all sections, see nature.com/documents/nr-reporting-summary-flat.pdf

Life sciences study design

All studies must disclose on these points even when the disclosure is negative.

Sample size	No sample size calculation was performed. We included n = 56 for electroencephalography with transcranial magnetic stimulation data and n = 49 for resting-state electroencephalography data.
Data exclusions	No data was excluded.
Replication	For a generalized model, we used the leave-one subject-out (LOSO) approach, which means each subject was tested, whereas other subjects except for one subject included in the testing phase were randomly used in the training phase. This LOSO approach is independent, so all attempts at replication were successful In this setting.
Randomization	All subjects performed the same study design, so randomization was not applicable here.
Blinding	All subjects performed the same study design, so blinding was not applicable here.

Reporting for specific materials, systems and methods

We require information from authors about some types of materials, experimental systems and methods used in many studies. Here, indicate whether each material, system or method listed is relevant to your study. If you are not sure if a list item applies to your research, read the appropriate section before selecting a response.

Materials & experimental systems

n/a	Involved in the study	
x		Antibodies
x		Eukaryotic cell lines
x		Palaeontology and archaeology
x		Animals and other organisms
	x	Human research participants
x		Clinical data
x		Dual use research of concern

Methods

n/a	Involved in the study	
x		ChIP-seq
x		Flow cytometry
x		MRI-based neuroimaging

Human research participants

Policy information about studies involving human research participants

Population characteristics	The sleep dataset and the anesthesia dataset included six healthy subjects (5 males, age 23.7 ± 3.2 years) and sixteen healthy subjects (8 males, age 18–28 years), respectively. For patients with severe brain injury and disorder of consciousness, we used the data of 15 patients with unresponsive wakefulness syndrome (8 males, 9 traumatic brain injuries, time since injury 8.0 months (1–47), age 39.3 ± 23.5 years), 15 patients in minimally conscious state (MCS) (11 males, 7 traumatic brain injuries, time since injury 64.8 months (1–343), age 40.5 ± 19.0 years), and four non-behavioral MCS patients (2 males, 3 traumatic brain injuries, time since injury 18.5 months (3–52), age 36.3 ± 10.5 years).
Recruitment	For sleep data, the inclusion criteria included (i) between 18 and 75 years of age and (ii) in good general healthy. The exclusion criteria was follows: (i) neurological, psychiatric, mood, and sleep disorders, (ii) contraindications for TMS (e.g., history of seizures), and (iii) psychotropic medication.<br>For anesthesia data, the inclusion criteria included (i) over 18 years of age and (ii) stability of vital parameters (healthy). The exclusion criteria was follows: (i) neurological, cardiovascular, psychiatric, and mood disorders, (ii) contraindications for TMS (e.g., history of seizures, metal implants such as a pacemaker), and (iii) medical conditions that were incompatible with the anesthesia and/or the TMS procedure.<br>For brain-injured patients data and added data, the inclusion criteria for all patients included (i) older than 18 years and (ii) diagnosis of disorders of consciousness following a severe acquired brain injury. The exclusion criteria for patients were as follows: (i) patients having significant neurological, neurosurgical, or psychiatric disorders prior to the brain injury that leads to disorders of consciousness, (ii) patients having any contraindication to TMS–EEG or MRI (electronic implanted devices, active epilepsy, external ventricular drain), and (iii) patients who were not medically stable.
Ethics oversight	All subjects or their legal guardians gave written consent, and the study was approved by the University of Wisconsin Human Subjects Committee for sleep dataset and the Ethics Committee of the Medicine Faculty of the University of Liege for the anesthesia and patients datasets.

Note that full information on the approval of the study protocol must also be provided in the manuscript.
